# Diversity of Nonribosomal Peptide Synthetases Involved in the Biosynthesis of Lipopeptide Biosurfactants

**DOI:** 10.3390/ijms12010141

**Published:** 2010-12-30

**Authors:** Niran Roongsawang, Kenji Washio, Masaaki Morikawa

**Affiliations:** 1 Microbial Cell Factory Laboratory, Bioresources Technology Unit, National Center for Genetic Engineering and Biotechnology (BIOTEC), National Science and Technology Development Agency (NSTDA), Pathumthani 12120, Thailand; 2 Division of Biosphere Science, Graduate School of Environmental Science, Hokkaido University, Sapporo 060-0810, Japan; E-Mail: washi@ees.hokudai.ac.jp

**Keywords:** lipopeptide biosurfactants (LPBSs), nonribosomal peptides, nonribosomal peptide synthetases (NRPSs)

## Abstract

Lipopeptide biosurfactants (LPBSs) consist of a hydrophobic fatty acid portion linked to a hydrophilic peptide chain in the molecule. With their complex and diverse structures, LPBSs exhibit various biological activities including surface activity as well as anti-cellular and anti-enzymatic activities. LPBSs are also involved in multi-cellular behaviors such as swarming motility and biofilm formation. Among the bacterial genera, *Bacillus* (Gram-positive) and *Pseudomonas* (Gram-negative) have received the most attention because they produce a wide range of effective LPBSs that are potentially useful for agricultural, chemical, food, and pharmaceutical industries. The biosynthetic mechanisms and gene regulation systems of LPBSs have been extensively analyzed over the last decade. LPBSs are generally synthesized in a ribosome-independent manner with megaenzymes called nonribosomal peptide synthetases (NRPSs). Production of active-form NRPSs requires not only transcriptional induction and translation but also post-translational modification and assemblage. The accumulated knowledge reveals the versatility and evolutionary lineage of the NRPSs system. This review provides an overview of the structural and functional diversity of LPBSs and their different biosynthetic mechanisms in *Bacillus* and *Pseudomonas*, including both typical and unique systems. Finally, successful genetic engineering of NRPSs for creating novel lipopeptides is also discussed.

## 1. Introduction

Biosurfactants are biological surface-active compounds largely produced by a wide variety of microorganisms. They have environmentally friendly properties, such as low toxicity and high biodegradability. Biosurfactants are capable of lowering surface and interfacial tensions effectively and thus are potential substitutes for widely used chemically synthesized surfactants. Thus, biosurfactants show promise for use in agricultural, food, pharmaceutical, cosmetic, specialty chemical, and oil production industries as well as in bioremediation technology. The hydrophobic portion of these molecules is commonly made up of fatty acids (saturated, unsaturated, or hydroxylated), whereas the hydrophilic portion is usually composed of peptides or mono-, di-, or polysaccharides [1]. Due to the presence of hydrophobic and hydrophilic moieties within a single molecule, (bio)surfactants tend to migrate toward an interface with different degrees of polarity and hydrogen bonding, such as an air/water or oil/water interface [2].

Production of effective lipopeptide biosurfactants (LPBSs) was first reported from Gram-positive *Bacillus subtilis* IAM1213 [3]. Since then, various types of LPBSs with significant surface activity and/or anti-microbial activity have been isolated from other *Bacillus* strains [4–7]. Gram-negative *Pseudomonas* spp. also produce a variety of LPBSs [8–12]. Gene clusters encoding multi-modular nonribosomal peptide synthetases (NRPSs) for LPBS production have been cloned and characterized from these two genera, and demonstrate their different evolutionary lineages. The systematic modular organization of NRPSs allows structural alteration of lipopeptides by swapping domains or modules of NRPSs to create novel lipopeptides [13,14]. This article aims to summarize current knowledge of the structural diversity of LPBSs and their biosynthetic systems in the genera *Bacillus* and *Pseudomonas*. Current possibilities and limits in the modification of their biosynthetic genes for the synthesis of novel compounds are also discussed.

### 2. Diversity of LPBSs and NRPSs from *Bacillus* and *Pseudomonas*

**Figure 1:**
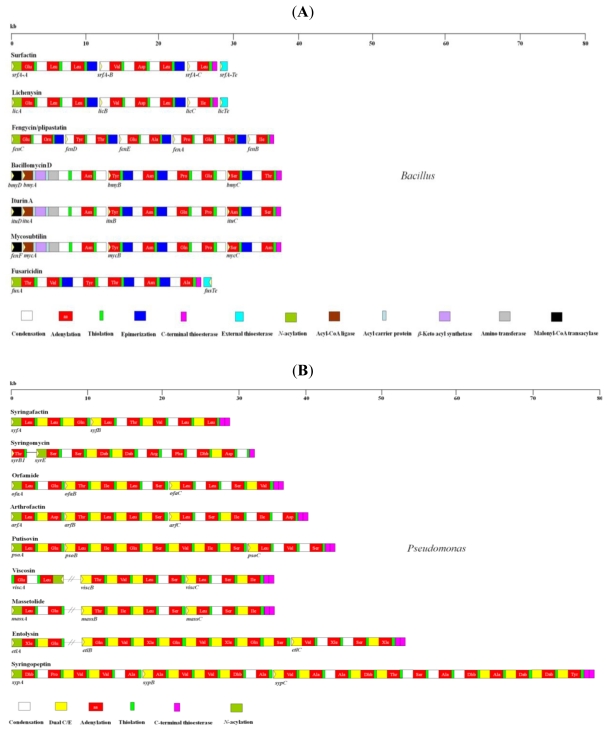
Multidomain organization of the gene clusters encoding NRPSs from *Bacillus* (**A**) and *Pseudomonas* (**B**).

*Bacillus* and *Pseudomonas* spp. produce a wide range of LPBSs (Tables 1, 2). These lipopeptides share similar amphipathic structures containing both a hydrophilic peptide portion and a hydrophobic fatty acid portion. Most of the LPBSs have a cyclic structure mediated by the linkage between a *C*-terminal peptide residue and either a β-hydroxy fatty acid, hydroxyl group of the peptide residue, or β-amino acid. NRPSs are multi-modular enzymes that recognize, activate, modify, and link the amino acid intermediates to the product peptide [15]. They are capable of synthesizing peptides that contain unusual amino acids including d-amino acids, β-amino acids, and hydroxy- or *N*-methylated amino acids. Biosynthesis of nonribosomal peptides occurs via the function of the catalytic unit, referred to as a module. The order of modules is usually co-linear to the product peptide sequences (Figure 1). Each module is composed of specific domains that are responsible for catalyzing different enzymatic activities.

The adenylation (A) domain is responsible for amino acid recognition and adenylation at the expense of ATP to form an acyl-adenylate intermediate. Then, the adenylated amino acid covalently binds to a phosphopantetheine carrier of the adjacent thiolation (T) or peptidyl carrier protein (PCP) domain. Peptide bond formation of two consecutively bound amino acids is catalyzed by the condensation (C) domain. Modification domains, such as the epimerization (E) domain, catalyze the conversion of l-amino acids to d-isomers, and they are typically associated with the module that incorporates d-amino acids. Lastly, cyclization and release of the product peptide are carried out by *C*-terminal thioesterase (Te) domain that is associated with a termination module. A number of gene clusters encoding NRPSs for LPBS biosynthesis in both *Bacillus* and *Pseudomonas* have been cloned and characterized (Figure 1). These gene clusters show striking similarities in the modular architecture of their repetitive catalytic units and assembly-line mechanism. However, several unique features have also been identified.

### 2.1. Bacillus

Most LPBSs from *Bacillus* can be classified into three families: surfactin, fengycin, and iturin [16]. There are several *Bacillus* strains that have the ability to produce three families of LPBSs simultaneously [17–20]. In addition to these three families, several other lipopeptides have also been identified in *Bacillus* species (Table 1). Characteristics of these LPBSs families and corresponding NRPSs are as follows.

#### 2.1.1. Surfactin and Lichenysin Synthetases

Surfactin and lichenysin are structurally related LPBSs produced by *B. subtilis* and *B. licheniformis* [3,7,21]. Several other forms of surfactin with amino acid variation at position 2, 4, and 7 have been reported [22]. Surfactin carries strong surfactant properties by reducing the surface tension of water from 72 to 27 mN/m at a critical micelle concentration (CMC) of 25–220 mg/L depending on its variants and determined conditions [3,23]. A surfactin-like compound termed lichenysin is at least a 2-fold more efficient biosurfactant than surfactin, probably due to the replacement of Glu1 by Gln1 [6,23]. Surfactin was first identified as an inhibitor of fibrin clot formation. It also exhibits anti-microbial, anti-tumor, anti-viral, and hemolytic properties [24]. Surfactin is required for the biofilm formation of producing cells [25,26], swarming motility [27,28], and fruiting body formation [29]. However, surfactin also inhibits biofilm formation of other bacteria by interfering with attachment of the cells to surfaces [30].

These LPBSs are usually a mixture of compounds with different lengths and types of fatty acid (FA), β-hydroxy FA (FA-β-OH), β-amino FA (FA-β-NH_2_) or guanidylated-β-OH FA (*g*FA-β-OH). The β-OH or β-NH_2_ group of FA forms an ester or peptide bond with the carboxyl group of the *C*-terminal amino acid. For fengycin, circulocins, fusaricidin, and kurstakin, the carboxyl group of the *C*-terminal amino acid is lactonised with the hydroxyl group of Tyr_3_, Thr_1_, Thr_1_, and Ser_4_, respectively.

The biosynthetic gene clusters of surfactin [69] and lichenysin [32], namely *srfA* and *lic*, are highly homologous and extend over 25 kb (Figure 1). They contain four open reading frames (ORFs), *srfA*-*A*/*licA*, *srfA*-*B*/*licB*, *srfA*-*C*/*licC*, and *srfA*-*Te*/*lic*-*Te*. The amino acid sequences of the first three ORFs are homologous to other NRPSs whereas the last ORF encodes a putative type II Te. SrfA-A/B/C and LicA/B/C are composed of three, three, and one module(s), and each ORF can be further subdivided into functional domains. SrfA and Lic bear six typical C-domains that catalyze amide bond formation. An additional C-domain, the *N*-acyl domain, is located at the *N*-terminal domain of the first module, suggesting that the first amino acid is initially *N*-acylated with a β-hydroxy fatty acid in this domain [32,70]. Recently, Kraas and coworkers (2010) have shown that the *N*-acyl domain in SrfA transfers CoA-activated 3-hydroxy fatty acid to the first T-domain where the *N*-terminal Glu is bound [71]. SrfA and Lic contain the conventional E-domains essential for the transformation of l-amino acids to d-amino acids and a *C*-terminal type I Te-domain that releases the final product. In addition to the *C*-terminal type I Te-domain, SrfA and Lic have an external type II Te protein, SrfA-Te/LicTe. A decreased production of surfactin (84%) is observed in the SrfA-Te mutant [72]. The external type II Te is involved in regenerating misprimed T-domains by removing short acyl chains from the 4′-phosphopantetheine cofactors and thereby regenerates functional NRPSs [73]. Moreover, a study has suggested that the type II Te also hydrolyzes incorrectly loaded amino acids that are not processed by the nonribosomal machinery [74]. SrfA-Te also functions as the thioesterase/acyltransferase that supports and stimulates the formation of β-hydroxymyristoyl-glutamate, an initiation substrate of surfactin synthesis [75,76].

#### 2.1.2. Fengycin Synthetase

Fengycin, also referred to as plipastatin when Tyr_3_ and Tyr_9_ is present as the l-and d-form, repectively. It is an anti-fungal antibiotic that inhibits filamentous fungi but is ineffective against yeast and bacteria. It is also capable of inhibiting phospholipase A_2_ and biofilm formation of several bacteria [4,37,77,78]. These types of lipodecapeptides are produced by various strains of *Bacillus* spp. and exhibit moderate surfactant activities [4,17,79]. Fengycin is expected to form a lactone between the hydroxyl group of l-Tyr_3_ and the *C*-terminal carboxyl group of l-Ile. Fengycin synthetase (Fen) contains five NRPS subunits: FenC (287 kDa), FenD (290 kDa), FenE (286 kDa), FenA (406 kDa), and FenB (146 kDa). Like SrfA and Lic, Fen is also composed of an *N*-acyl domain at the *N*-terminus of FenC, conventional E-domains, and a typical type I Te-domain. Fen assembles to form a co-linear chain ordered as FenC-Fend-FenE-FenA-FenB [80].

#### 2.1.3. Bacillomycin, Iturin, and Mycosubtilin Synthetases

The iturin family comprises bacillomycin, iturin, and mycosubtilin, which are cyclic lipoheptapeptides linked by a β-amino acid residue. Members of this family have strong antibiotic activity, moderate surfactant activity, and enhanced swarming motility [17,38,81]. The NRPS gene cluster of bacillomycin D (*bam/bmy*), mycosubtilin (*myc*), and iturin A (*itu*) is composed of four large ORFs [18,40,82,83]. *bam* and *bmy* are identical gene clusters found in *B. subtilis* AU195 and *B. amyloliquefaciens* FZB42, respectively. The gene encodes multifunctional hybrid enzymes of a fatty acid synthase, an aminotransferase, and peptide synthetases. The first ORF-*bmyD*, *ituD*, and *fenF*-encodes malonyl-CoA transacylase. The second ORF-*bmyA*, *ituA*, and *mycA*-encodes acyl-CoA ligase, acyl carrier protein (ACP), β-ketoacyl synthetase and aminotransferase domains before a conventional module of NRPS. The MycA loading module activates free fatty acids through an acyl-adenylate intermediate and loads on the adjacent ACP1 domain [84]. Meanwhile, FenF reveals broad acyl-substrate specificity and loads malonyl-CoA onto ACP2 in MycA [85]. The aminotransferase domain catalyzes the transfer of an amino group to the β-position of the growing acyl chain [86]. The resulting β-amino fatty thioester is then presumably passed on to the third and fourth ORFs that encode four and two functional modules of a typical NRPS, respectively. Mycosubtilin and iturin A have almost the same structure except that d-Ser_6_ and l-Asn_7_ residues in mycosubtilin are inverted to d-Asn_6_ and l-Ser_7_ in iturin A. Amino acid sequence homology between the two A-domains for Ser and Asn in the two synthetases is high, suggesting that an intragenic domain change occurred in either Myc or Itu synthetase to evolve a counterpart gene [82].

#### 2.1.4. Fusaricidin Synthetase

Fusaricidin is a unique hexapeptide linked to guanidylated β-hydroxyl fatty acid that possesses a potent anti-fungal activity produced by *Paenibacillus polymyxa* PKB1 (formerly called *Bacillus polymyxa*). It is a candidate for a biocontrol medicine used to treat blackleg disease [44]. Fusaricidin synthetase (FusA) comprises six NRPS modules that are encoded by a single ORF. The second, fourth, and fifth, modules of FusA incorporate d-amino acids and carry E-domains. However, no E-domain was detected in the sixth module that would incorporate d-Ala [44].

To date, three different mechanisms have been reported for incorporation of d-amino acids in the LPBS products. In the typical NRPS system in Gram-positive bacilli, an E-domain responsible for epimerization of l-amino acids to the d-forms is located downstream of d-amino acid-incorporating modules. A second mechanism is the direct incorporation of d-amino acids by the respective A-domains. This system is found in eukaryotic fungal NRPS systems such as cyclosporine and HC-toxin [87,88]. A third system for incorporating d-amino acid is a novel type of C-domain with dual epimerization and condensation activities (C/E domain), which has recently been identified in several NRPSs in both actinomycete and Gram-negative pseudomonads [89].

Regarding FusA synthetase, two different mechanisms for incorporation of d-amino acids are employed. Conversion from l- to d-amino acid in the three modules of FurA synthetase is mediated by conventional E-domains, whereas direct incorporation of d-Ala is found in the last module that does not contain the E-domain or C/E domain [44]. Thus, horizontal gene transfer has potentially occurred between Gram-positive bacterial and fungal NRPS genes.

### 2.2. Pseudomonas

*Pseudomonas* also produces a variety of cyclic lipopeptides. Recently, lipopeptides of pseudomonads have been classified into six groups: viscosin, syringomycin, amphisin, putisolvin, tolaasin, and syringopeptin [90]. In addition to these six main LPBS groups, other lipopeptides have been also identified in *Pseudomonas* species, but no cyclic lipopeptides linked by a β-amino acid residue have been reported (Table 2).

#### 2.2.1. Syringomycin Synthetase

Syringomycin is a phytotoxin and a key determinant of *Pseudomonas syringae* B301D virulence. It has moderate surfactant activity with a CMC of 1250 mg/L and minimum surface tension of 33 mN/m [91]. Syringomycin is synthesized by two NRPSs (SyrB1, SyrE) and three modifying protein systems (SyrB2, SyrC, SyrP). SyrB1 and SyrE do not follow the co-linearity rule and also lack E-domains. Eight modules for the first eight amino acids in SyrE are arranged in a line, but the ninth module (SyrB1), which is necessary for incorporation of the last amino acid (l-Thr_9_), is located in the upstream region [51]. This observation suggests that absolute co-linearity is not essential for NRPS synthesis, which is similar to SrfA data [92]. l-Thr_9_ is activated and loaded by SyrB1 and is then chlorinated to 4-Cl-l-Thr by the non-heme Fe(II) halogenase SyrB2 [93]. This intermediate is transferred from the T-domain of SyrB1 to SyrE by aminoacyltransferase SyrC to form the final product [94]. Hydroxylation of Asp at module 8 is catalyzed by SyrP, whose gene is located upstream of *syrB1* [95]. Although three amino acid residues in syringomycin are in the d-form, the E-domain is not associated with the modules incorporating the respective d-amino acids. However, Balibar and coworkers (2005) demonstrated that SyrE contains unique dual C/E domains, which contribute to the conversion of l-amino acids to the d-form [89].

#### 2.2.2. Syringopeptin Syntheatase

*P. syringae* B301D also produces another class of lipodepsipeptide phytotoxins called syringopeptin. Syringopeptin contains a larger peptide moiety than syringomycin, with 22 or 25 amino acid residues, and is one of the largest LPBS ever reported. Syringopeptin has a CMC of 820 mg/L and reduces surface tension to 40.2 mN/m [91]. Three NRPSs, SypA, SypB, SypC, are involved in the biosynthesis of syringopeptin. The order and number of the modules are co-linear to the amino acid sequence of syringopeptin SP22. SypA/B/C represents the largest NRPSs among those reported for prokaryotes. Similar to Syr synthetase, no E-domain is present in Syp synthetase, despite the presence of several d-amino acids. In contrast to the Syr synthetase, SypC contains two unique *C*-terminal Te-domains predicted to catalyze the release and cyclization of syringopeptin [96].

#### 2.2.3. Arthrofactin Synthetase

Arthrofactin is a cyclic lipoundecapeptide produced by *Pseudomonas* sp. MIS38, which was initially misidentified as *Arthrobacter* sp. [56], and belongs to the amphisin group. The molecule is cyclized through the formation of an ester bond between the carboxyl group of the *C*-terminal Asp and the β-hydroxyl group of d-*allo*-Thr [97]. Arthrofactin is one of the most effective cyclic LPBSs; it reduces the surface tension of water from 72 to 24 mN/m with a CMC of 13.5 mg/L [56]. Arthrofactin appears to be essential for swarming activity and inhibits initial attachment of the planktonic cells in biofilm formation [98]. Several arthrofactin-like compounds with remarkable biosurfactant and antifungal properties or an enzyme inhibitor have been reported from *Pseudomonas* spp. [9–12,57]. Like arthrofactin, amphisin is involved in swarming motility of *Pseudomonas* sp. DSS73 [99].

**Figure 2:**
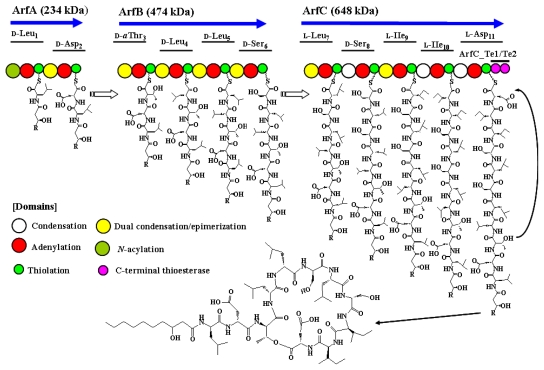
The arthrofactin biosynthesis assembly line.

Biosynthesis of arthrofactin is catalyzed by the arthrofactin synthetase (Arf), which consists of three NRPS protein subunits, ArfA (234 kDa), ArfB (474 kDa), and ArfC (648 kDa), which contain two, four, and five functional modules, respectively (Figure 2) [98]. An additional C-domain was identified in the first module of ArfA, suggesting that the first amino acid could be initially acylated with a fatty acid. Site-directed mutagenesis changing the histidine residue of conserved core motif (H**H**XXXDG) to alanine impairs arthrofactin production [100]. This result suggested that the first C-domain is essential for biosynthesis of lipopeptide. Indeed, the β-hydroxydecanoyl thioester may be coupled to the activated leucine by the action of this C-domain to yield β-hydroxydecanoyl-l-Leu as the initial intermediate. A phylogenetic tree showed that the first C-domain of Arf belongs to *N*-acyl groups that use fatty acyl-CoA as their starter unit [70]. Although seven of the 11 amino acid residues in arthrofactin are in the d-form, Arf contains no E-domains, as found in syringomycin and syringopeptin [98]. The A-domain of d-Leu_1_ specifically recognizes only l-Leu *in vitro*. Based on these observations, we initially hypothesized that an external racemase may be responsible for incorporation of the d-amino acids in arthrofactin. Different amino acid sequences downstream of a conserved core motif [FFELGGHSLLA(V/M)] in the T-domains were expected to reflect the recognition by external racemase. However, Balibar and coworkers later demonstrated that Arf contains unique dual C/E domains, which contribute to the conversion of l-amino acids to the d-form [89]. This novel C/E domain is cryptically embedded with the C-domain located downstream of the d-amino acid–incorporating modules. Dual C/E domains can be recognized by an elongated His motif (HHI/LXXXXGD). This feature was also identified in the Syr and Syp synthetases. Another unique characteristic of Arf is the presence of *C*-terminal tandem Te-domains like syringopeptin. By site-directed mutagenesis, the first Te-domain (ArfC-Te1) was shown to be essential for the completion of macrocyclization and the release of the final product. The second Te-domain (ArfC-Te2) was suggested to be involved in the evolution of Arf to improve the macrocyclization efficiency [101]. Moreover, we found that the gene encoding putative ArfA/B/C exists in the genome sequence of *Pseudomonas fluorescens* Pf0-1 (YP_347943/YP_347944/YP_347945) [102]. Arf represents a novel NRPS architecture that features tandem Te-domains and dual C/E domains. Interestingly, another type of NRPS involved in biosynthesis of a siderophore, pyoverdine, was also identified in arthrofactin-producing *Pseudomonas* sp. MIS38. A gene encoding NRPS for the chromophore part of pyoverdine contains a conventional E-domain [102]. This observation suggests that different NRPS systems with dual C/E domains and a conventional E-domain are both functional in *Pseudomonas* spp.

#### 2.2.4. Viscosin and Massetolide Synthetases

Viscosin and massetolide are structurally related lipononapeptides produced by *P. fluorescens* SBW25 and *P. fluorescens* SS101, respectively [46,48]. Viscosin has significant surfactant activity by reducing surface tension of water to 28 mN/m with a CMC of 10–15 mg/L and forms stable emulsions [46,103]. It also inhibits migration of a metastatic prostate cancer cell line without visible toxicity [103]. In addition, viscosin and massetolide are required for biofilm formation and swarming motility of *Pseudomonas* cells [46,48]. Viscosin/massetolide is synthesized by NRPS systems that are encoded by three large ORFs, termed *viscA*/*massA*, *viscB*/*massB*, and *viscC*/*massC*. The *viscA*/*massA* gene is not clustered with the latter genes, but is located at a different locus of the *Pseudomonas* genome. The distance between *viscA*/*massA* and the latter genes is more than 1.5 MB. Analysis of the amino acid sequences revealed two modules in ViscA/MassA, four modules in ViscB/MassB, and three modules in ViscC/MassC. Each module bears A-, T-, and C-domains like other NRPSs. However, none of the five d-amino acid-incorporating modules possesses a cognate E-domain, but contains a C/E domain similar to Arf. Tandem Te-domains were also identified in the last ViscC/MassC module and are likely to be functional for the biosynthesis of both lipopeptides as was shown for the two Te-domains in Arf [101]. Similar to other NRPSs involved in lipopeptide biosynthesis, the *N*-terminal C-domain in the first module is highly similar to the *N*-acyl domain and is presumably involved in *N*-acylation of the first amino acid.

#### 2.2.5. Orfamide Synthetase

Orfamide and its biosynthetic genes (*ofaA/B/C*) were discovered from the *P. fluorescens* Pf-5 genome using a genome isotope approach that employs a combination of genome sequence analysis and isotope-guided fractionation to identify the corresponding compounds [66]. Orfamide is a lipodecapeptide consisting of a β-hydroxy fatty acid linked to a 10-amino acid cyclic peptide in which five amino acids are in the d-form. Orfamide is essential for swarming activity and exhibits strong zoosporicidal activity, but it is not involved in biofilm formation [66]. Although orfamide is composed of 10 amino acids, its structure is most similar to the lipononapeptide viscosin, suggesting a common evolutionary lineage. It is also interesting that the gene structure, *ofaABC*, is rather similar to *arfABC* (Figure 1). Structural analysis of OfaA/B/C identified ten modules. The first NRPS, OfaA, consists of two modules with an *N*-acyl domain at its *N*-terminus. Four modules were identified in the second NRPS, OfaB, and the last NRPS, OfaC. Similar to other *Pseudomonas* NRPSs, no cognate E-domains are found in Ofa modules. Although five amino acid residues in orfamide are in the d-form, Ofa seems to contain a total of six dual C/E domains. This inconsistency suggests that the NRPS system is more complex than previously thought. Tandem Te-domains were found in the *C*-terminus of OfaC, similar to several NRPSs from other *Pseudomonas* species.

#### 2.2.6. Putisolvin Synthetase

Putisolvin is a LPBS synthesized by *P. putida* PL1445, which was isolated from soil heavily contaminated with polycyclic aromatic hydrocarbons. Putisolvin is a cyclic lipododecapeptide consisting of a 12-amino acid peptide linked to a hexanoic lipid by an ester linkage between the ninth serine residue and the *C*-terminal carboxyl group [58]. Putisolvin inhibits biofilm formation of other bacteria and exhibits zoosporicidal and antifungal activities [58,104]. Three genes (*psoA*, *psoB*, and *psoC*) were identified and shown to encode NRPS involved in putisolvin biosynthesis [105]. PsoA, PsoB, and PsoC contain two, seven, and three functional modules, respectively. The *C*-terminus in PsoC carries putative tandem Te-domains. Both domains harbor a highly conserved signature sequence (GXSXG) and the catalytic triad residues of Te-domains. Nine of the 12 amino acids are in the d-form, but no conventional E-domains were identified [105]. Analysis of specific sequence motifs in the T-domains suggested that the first nine T-domains in Pso synthetase are responsible for transferring d-amino acids [98]. Amino acid sequence analysis of the C-domains indicated that dual C/E domains are organized downstream of the first nine modules. Prediction of A-domain substrate specificity in the eleventh module indicates its preference for Val over Leu or Ile, which correlates well with the production ratios of putisolvin I and II [105].

#### 2.2.7. Syringafactin Synthetase

Syringafactin is a novel linear lipooctapeptide produced by *P. syringae* pv. *tomato* DC3000. It contains an eight-amino acid linear peptide linked to a β-hydroxy fatty acid. The Val_4_ residue can be substituted with Leu or Ile. Syringafactin shows surfactant activity and is essential for the swarming motility of the producing strain, but its contribution to the pathogenicity has not been tested [68]. Syringafactin biosynthetic genes were identified from mining the *P. syringae* pv. *tomato* DC3000 genome. The gene clusters *syfA* and *syfB* encode three and five NRPS modules, respectively. The *N*-acyl domain present in the initiating module of SyfA indicated that syringafactin would contain an *N*-terminal fatty acid chain. SyfB contains tandem Te-domains at the *C*-terminus. The d/l-configuration of each residue has not been determined. However, based on the location of the dual C/E domains that are typically located downstream of d-amino acid–incorporating modules, the structure of syringafactin should be fatty acyl-d-Leu_1_-d-Leu_2_-d-Gln_3_-Leu_4_-d-Thr_5_-Val_6_-d-Leu_7_-Leu_8_, which differs from a previous report [68]. The *N*-terminal C-domain of SyfA shows the highest level of amino acid sequence similarity with the *N*-terminal C-domain of ArfA. It seems likely that protein domains corresponding to the first three modules of the arthrofactin NRPS are absent in syringafactin NRPS. This observation suggests that the syringafactin NRPS system in *P. syringae* pv. *tomato* DC3000 evolved from the arthrofactin system, after which three modules of the arthrofactin NRPS were deleted, resulting in the fusion of the *N*-terminus of ArfA with a portion of ArfB. Importantly, the deleted modules include the module that incorporates the threonyl residue that forms the ester linkage involved in cyclization of arthrofactin. Indeed, the structure of syringafactin is reported to be a linear form.

#### 2.2.8. Entolysin Synthetase

Entolysin is a cyclic lipotetradecapeptide produced by an entomopathogenic bacterium *Pseudomonas entomophila*. This bacterium is able to infect and effectively kill various insects, and it is closely related to the saprophytic soil bacterium *P. putida*. Entolysin has a relatively small cyclic peptide moiety in which a lactone ring is formed between the tenth and the last amino acid. Entolysin is required for swarming motility and exhibits hemolytic and surfactant activity as described for other lipopeptides, but it does not participate in the virulence of the producing strain for killing *Drosophila* [65]. Three genes encoding entolysin synthetases were identified (*etlA*, *etlB*, and *etlC*). The deduced amino acid sequences are similar to other NRPSs and closely related to Pso synthetase in *P. putida* PCL1445. The *etlA* gene is not physically linked with *etlB* and *etlC* in the *P. entomophila* genome. This organization has also been reported for the viscosin and massetolide gene clusters. EtlA, EtlB, and EtlC comprise two, eight, and four functional modules of NRPS, all of which correspond to the number of amino acid residues in the product peptide. These modules are composed of typical domains. However, no cognate E-domains have been identified in EtlA/B/C. Amino acid sequence analysis indicated that all of the C-domains but C12 and C13 could function as dual C/E domains. In addition, the first C-domain of EtlA is similar to the *N*-acyl domain of other lipopeptides, and tandem Te-domains were identified in the *C*-terminus of EtlC [65].

## 3. Gene Regulation in *Bacillus*

**Figure 3:**
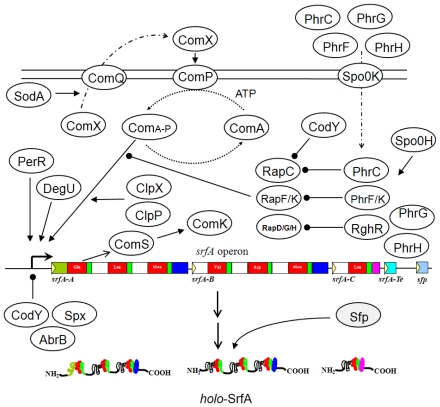
The model of gene regulation involved in surfactin biosynthesis. Closed-head arrows indicate positive regulation whereas closed circles indicate negative regulation.

Gene regulation of LPBSs produced by *Bacillus* spp. has been most intensively investigated within the surfactin biosynthesis system (Figure 3). Expression of the gene *srfA* is controlled by several peptide pheromones including ComX and Phr [106]. *B. subtilis* encodes eight Phr peptides (PhrA, PhrC [CSF], PhrE, PhrF, PhrG, PhrH, PhrI, and PhrK) and 11 aspartyl-phosphate phosphatase proteins (RapA to RapK). Each Phr peptide inhibits the activity of cotranscribed Rap protein. RapC, RapF, and RapK act as negative regulators of *srfA* [107]. ComX interacts with the membrane-bound histidine kinase ComP, which autophosphorylates upon stimulation and then transfers its phosphate to a serine residue in the response regulator ComA. The phosphorylated ComA binds to ComA boxes (T/GCGG-N4-CCGCA) upstream of the *srfA* promoter as a tetramer and initiates transcription of *srfA* [108,109]. Recently, it was found that ComA binds to a degenerate tripartite sequence consisting of three recognition elements (RE). RE1 and RE2 contain the inverted repeats previously characterized as part of the ComA-boxes. Meanwhile, RE3 is located downstream of RE1 and RE2 with a consensus sequence identical to that of RE1 [110]. In addition, mutation at three non-aspartate amino acids in the *N*-terminal portion of ComA decreases surfactin production [111]. These three amino acids may be involved in the phosphorylation mechanism. It was previously reported that glucose can stimulate the transcription of *comA* and consequently increases the expression of *srfA* [112,113]. Induction of *srfA* requires the oligopeptide permease Spo0K, which is involved in PhrC import [114]. On the other hand, expression of *srfA* is downregulated upon treatment with H_2_O_2_; this evidence led to identification of the H_2_O_2_ stress responsive regulator PerR. PerR positively regulates *srfA* expression by binding to PerR boxes located in the upstream region of ComA boxes in which H_2_O_2_ inhibits the DNA-binding activity of PerR [115]. Furthermore, the chaperon subunit ClpX and protease ClpP are required for the transcription of *srfA* at a step that follows ComP-dependent activation of ComA [116]. An additional transcription factor DegU also functions as a positive regulator for *srfA* transcription [117].

Overexpression of RapD, RapG, and RapH inhibits *srfA* transcription, and production of these Rap proteins is suppressed by RghR [118,119]. Mutation in *sodA*, which encodes superoxide dismutase, inhibits transcription of the comQXP quorum-sensing locus, thereby preventing *srfA* expression [120]. At high concentrations of amino acids such as Ile, Leu, and Val, CodY represses transcription of *srfA* by interacting specifically with the *srfA* promoter [121]. Like CodY, AbrB also negatively regulates *srfA* transcription. Expression of *srfA* is also repressed by the RNA polymerase-binding protein Spx [122]. On the other hand, the Spx-RNA polymerase interaction is required for positive transcriptional control of genes in response to thiol-oxidative stress [123]. Furthermore, 4′-phosphopantetheinyl transferase (Sfp/PPTase) is required for the activation of SrfA enzymes by converting the inactive *apo*-forms of the T-domains to the active *holo*-forms [124]. An acyltransferase SrfA-Te is also required in the initial step of transferring a hydroxyl fatty acid to the first amino acid in the peptide. The surfactin self-resistance protein, YerP, is required for surfactin exportation [125].

Regulation of the fengycin/plipastatin genes is positively controlled by DegQ, an enhancer of extracellular protease production [126]. *degQ* is a pleiotropic regulatory gene that controls the production of several hydrolytic enzymes [127]. Production of plipastatin is severely reduced in the *degQ* mutant, but no significant change is observed in surfactin production. A Sfp-like protein, Lpa-8, is also required for plipastatin production in *B. subtilis* YB8 [128]. Overexpression of *degQ* in *B. subtilis* 168 expressing *lpa*-*8* yields a 10-fold increase in plipastatin production [126]. Recently, transcription analysis of *fen* in *B. subtilis* F29-3 demonstrated that RNA polymerase binds to the Aand T-rich sequences, called the UP element, which is located upstream of the *fen* promoter [129].

Gene regulation of the iturin family was first demonstrated within mycosubtilin-producing *B. subtilis* ATCC6633. Expression of the *myc* operon is independent of ComA, but still seems to be regulated via quorum sensing, as PhrC strongly stimulates expression. The sigma H factor, Spo0H, also influences expression of the *myc* operon, and addition of PhrC to the culture medium compensates for loss of Spo0H expression. Finally, the transition state regulator AbrB represses expression of *myc*, as deletion of *abrB* results in increased *myc* expression [130]. Further information regarding the gene regulation was obtained from the study of the *bmy* operon produced using the *B. amyloliquefaciens* FZB42. Expression of *bmy* is dependent on a single sigma A factor-dependent promoter and is positively controlled by the small regulatory protein DegQ, similar to *fen*. Similar to *srfA*, the global regulators DegU and ComA are required for the full transcriptional activation of *bmy*. DegU plays a key role because it binds directly to two sites located upstream of the *bmy* promoter. Moreover, post-transcriptional regulation of bacillomycin production is also suggested for both DegU and a transmembrane protein, YczE [131]. Like other lipopeptide synthetases in *Bacillus*, the Sfp-like protein, Lpa-14, is also required for iturin production [132]. Mutation of the Sfp-encoding gene simultaneously prevents *B. amyloliquefaciens* FZB42 from producing bacillomycin, fengycin, and surfactin [131].

## 4. Gene Regulation in *Pseudomonas*

**Figure 4:**
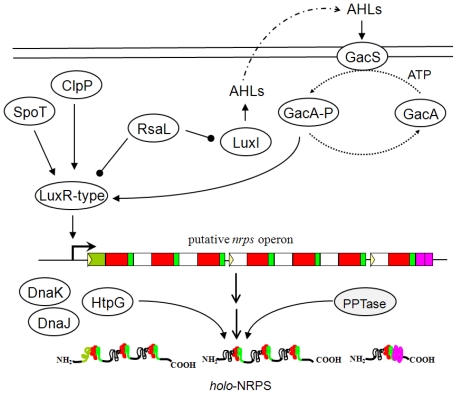
The model of gene regulation involved in lipopeptide biosynthesis by *Pseudomonas*. Closed-head arrows indicate positive regulation whereas closed circles indicate negative regulation.

Similar to ComP/ComA in *Bacillus*, a two-component system has been identified as the master transcriptional regulation system in *Pseudomonads* (Figure 4). Typically, this system consists of a sensor kinase GacS and response regulator GacA. GacS was first described in *P. syringae* pv. *syringae* B728a as an essential factor for lesion manifestation. Meanwhile, GacA was first identified as a global activator of antibiotic and cyanide production in *P. fluorescens* CHA0 [133]. It is proposed that upon interaction with the signal(s), GacS is activated by autophosphorylation and then GacA acts as a phosphoryl acceptor. After trans-phosphorylation, GacA activates transcription of the regulatory gene, which in turn controls the expression of target genes [133]. Based on bacterial two-hybrid analysis, the entire GacA molecule is necessary for GacA interaction with itself or GacS [134]. The GacS/GacA system positively controls the expression of genes required for the synthesis of lipopeptides (syringomycin, amphisin, putisolvin, and entolysin) because mutation in either gene impairs lipopeptide production [65,135–138]. A quorum-sensing system that triggers GacA/GacS phosphorylation during high cell density is essential for biosynthesis of the lipopeptide putisolvin [139]. In Gram-negative bacteria, the quorum-sensing system largely relies on the interaction of signaling molecule *N*-acyl homoserine lactones (AHLs) that are synthesized via LuxI protein with the transcriptional regulator LuxR. The quorum-sensing system in *P. putida* PCL1445 is composed of LuxI-homologous PpuI, LuxR-homologous PpuR*,* and RsaL. Expression of the genes *ppuI* and *ppuR* are required for the biosynthesis of AHLs, and mutation of these genes reduces putisolvin production. Meanwhile, overproduction of AHLs and putisolvin is observed in the *rsaL* mutant. This observation suggests that RsaL acts as a repressor of PpuI and PpuR. In contrast, biosynthesis of the lipopeptides massetolide, amphisin, and syringomycin is not regulated by AHL-based quorum sensing [48]. Downstream of the Gac system, the cognate transcriptional regulator LuxR regulates the production of several lipopeptides that bind to the operator of the NRPS genes. LuxR protein contains a DNA-binding helix-turn-helix motif in its *C*-terminal region. In *Pseudomonas* sp. MIS38, the LuxR-type transcription factor, ArfF, positively controls transcription of the gene *arf* [140]. LuxR-type protein is also implicated in the biosynthesis of entolysin, putisolvin, and syringafactin, and mutation of this gene results in the loss of lipopeptide production [65,68,105,141]. Two types of LuxR-type proteins are involved in biosynthesis of syringomycin, syringopeptin, and viscosin. SalA and SyrF are the LuxR-type proteins responsible for the production of two lipopeptides, syringomycin and syringopeptin, in *P. syringae* pv. *syringae* B301D. SalA is suggested to control transcription of SyrF, an apparent homolog of ArfF, and SyrF binds and trans-activates the target NRPS promoter [142]. Mutation of two LuxR-type proteins in *P. fluorescens* SBW25, ViscAR and ViscBCR, results in the reduction of NRPS gene transcription and loss of viscosin production [143]. Recently, heat shock proteins were shown to regulate biosynthesis of putisolvin and arthrofactin [140,141]. Mutation in the gene encoding DnaK, a HSP70 class heat shock protein, impairs putisolvin production in *P. putida* PCL1445. Together with DnaJ, DnaK regulates putisolvin synthesis at low temperature. Elimination of arthrofactin synthesis was identified following mutation of the gene encoding HtpG, a HSP90 class heat shock protein. However, normal expression of the arthrofactin synthetase genes is retained in the HtpG mutant. Thus, HtpG appears to be involved in the proper folding of positive transcription factors or in the assembly of the NRPS complex. The serine protease ClpP regulates massetolide biosynthesis in *P. fluorescens* SS101 via LuxR transcriptional regulators, and expression of ClpP is independent of regulation by the GacS/GacA system [144]. In contrast to surfactin synthesis, the chaperon subunit ClpX is not involved in the production of massetolide, and its gene is transcribed independently of *clpP*. Random mutagenesis in *Pseudomonas* MIS38 also led to identification of SpoT as a new regulator of arthrofactin synthesis [140]. Mutation in the SpoT-encoding gene prevents MIS38 from producing arthrofactin. SpoT is a (p)ppGpp synthetase/hydrolase responsible for cellular metabolism during nutritional starvation [145]. Epistasis analysis revealed that *spoT* positively regulates arthrofactin biosynthesis through *arfF* and *arfB*. Post-translational modification of NRPS in pseudomonads is also catalyzed by PPTase [146–148].

Exportation of lipopeptide in pseudomonads requires ATP-binding cassette (ABC) transporter systems, and the ABC transporter genes are then clustered together with a synthetase gene [48,65,68,98,105]. Exportation of syringomycin and syringopeptin requires two transporter systems known as SyrD and PseABC. SyrD is proposed to function as an ATP-driven efflux pump. Only trace quantities of both lipopeptides are produced by the *syrD* mutant, and the cells show significantly lower virulence [149, 150]. On the other hand, the tripartite ABC-type efflux transporter PseABC is homologous to the resistance-nodulation-cell division, RND, efflux system. Mutation in each of the genes *pseABC* results in a 40 to 60% decrease in both syringomycin and syringopeptin production [151]. The transporter system for arthrofactin was recently characterized in *Pseudomonas* MIS38 [152]. The genes encoding a putative periplasmic protein (ArfD) and a putative ABC transporter (ArfE) are located downstream of the *arf* gene cluster. Arthrofactin production is temporarily reduced in both mutants, but it eventually reached a similar level to that of MIS38 after 12 h cultivation. Furthermore, exportation of arthrofactin is almost completely blocked by ABC transporter inhibitors. This suggests that multiple ABC transporter systems can export arthrofactin and that accumulation of arthrofactin is toxic to the cells. Two genes that are predicted to encode homologs of the ABC transporters MacA and MacB in *E. coli* are located downstream of the putisolvin and entolysin synthetase genes [153]. Analysis of *macA* and *macB* mutations in *P. putida* PCL1445 shows a 70% decrease in putisolvin production [105]. Meanwhile, *macA* and *macB* mutations in *P. entomophila* result in almost a complete loss of entolysin production [65]. Therefore, we hypothesize that multiple ABC transporter systems generally play a role in transportation of LPBSs in pseudomonads.

## 5. Genetic Engineering of NRPS to Create Novel Products

A large number of LPBSs are synthesized by NRPSs that share similar modular architecture of their repetitive catalytic units and a similar assembly-line mechanism. However, recent genome analysis of the lipopeptide synthetases from both *Bacillus* and *Pseudomonas* strains revealed novel NRPS architecture that does not completely conform to the co-linearity rule, which encompasses multifunctional hybrid enzymes that can directly incorporate d-amino acid residues. In addition, most of the recently identified lipopeptide synthetases in *Pseudomonas* spp lack the cognate E-domains, but contain a dual C/E domain and also feature tandem Te-domains. These variations demonstrate natural versatility in evolving complex pathways for lipopeptide biosynthesis and may allow for artificial alteration of the protein template with the aim of reprogramming it to create novel compounds with improved properties.

Recently, new strategies for engineering NRPSs have been developed. The first successful report examined exchanging or replacing the minimal module (A- and T-domains) with the last Leu_7_ module within SrfA-C, a single-module with the simplest structure [14]. This module was deleted and replaced with several bacterial and fungal A/T-domains by homologous recombination. Construction of the gene encoding hybrid SrfA leads to the production of surfactin variants that retain their activity (Table 3). Using the same strategy, A/T-domain exchange was applied to multi-modular SrfA. Swapping of the Leu_2_ module results in an altered product whose peptide chain is shorter than that of wild-type [154]. However, this minimal module replacement resulted in a very low yield of variants. Therefore, Yakimov and coworkers (2000) developed a whole-module replacement within SrfA. A complete set of C/A/T-domains of the lichenysin A synthetase Gln_1_ module was introduced into the Glu_1_ module of SrfA. An altered product, surfactin [Gln_1_], was produced by the recombinant *B. subtilis* at a level similar to that of wild-type surfactin and it also exhibits stronger surface activity than surfactin [155]. The SrfA could also be modified by translocation of the *C*-terminal Te-domain to the end of the internal domains, thus resulting in new linear surfactin analogs [156]. Furthermore, the use of recombinant Te-domain to catalyze regiospecific cyclization of synthetic peptides or lipopeptides *in vitro* is a powerful approach for generating libraries of novel compounds with improved properties [157,158]. Another powerful approach for the genetic manipulation of NRPS templates is the directed mutation of the substrate specificity within the A-domain based on its specificity-conferring code [159]. Specificity-conferring codes of the Asp_5_ module within SrfA were adapted for the recognition of Asn. The engineered *B. subtilis* produces the new lipoheptapeptide, surfactin [Asn_5_] [13]. Along the same line, directed mutation of the Asp_7_ A-domain to Asn_7_ within CDA synthetase from *Streptomyces coelicolar* leads to production of the expected lipoundecapeptide containing Asn_7_ and an unexpected linear lipohexapeptide intermediate [160]. A reduction or increase in the number of peptide residues is an alternate approach to generate structural diversity of lipopeptides and glycopeptides. Deletion of an entire Leu_2_ module in SrfA causes secretion of the predicted surfactin variant with a smaller cyclic peptide. Furthermore, a novel lipohexapeptide is produced with a significantly high yield and enhanced antimicrobial activities [161, 162]. Amino acid insertion by module extension has been performed within the vancomycin-type glycopeptide antibiotic, balhimycin. Insertion of an entire d-hydroxyphenylglycine module into the balhimycin assembly line between modules 4 and 5 results in an elongated octapeptide product [163]. *C*-terminally truncated hexa- to di-peptide metabolites are also detected. An alternative approach was examined based on the function of communication-mediating (COM) domains that play an important role in the intermolecular communication within the NRPS system [164]. Swapping of COM domains have been exploited within SrfA and allow for biosynthesis of surfactin variants with different peptide chain lengths [165]. Using plasmid and transposon mutation of the *srfA* operon at specific and random positions, various intermediates from lipodipeptides to lipohexapeptides were identified by whole-cell MALDI-TOF MS analysis [166]. Although several NRPSs involved in lipopeptide production in pseudomonads have also been characterized, there is limited information available for rational modification of their NRPS modules *in vivo*. Utilizing a complementation method, Ackerley and Lamont (2004) examined the possibility for engineering pyoverdine synthetase (PvdD) in *P. aeruginosa* PAO1 to generate novel pyoverdine analogs. Introduction of the Thr-incorporating module from other species into the *pvdD* mutant restores pyoverdine production [167]. Recently, the researchers at Cubist Pharmaceuticals, Inc. have shown how these approaches can also be used for the production of lipopeptide variants by *Streptomyces* spp. with improved antibiotic activities [168,169].

## 6. Conclusion

Gram-positive *Bacillus* and Gram-negative *Pseudomonas* strains produce a variety of lipopeptides with remarkable surface and biological activities. In contrast to the structural diversity of these lipopeptides, their biosynthetic mechanism is basically conserved. They are synthesized nonribosomally by a mega-peptide synthetase unit, NRPS, which is composed of several cooperating multifunctional modules, each capable of performing one cycle of peptide elongation. To become an active form, they are post-translationally modified by a PPTase and properly assembled by DnaJ/K and HtpG proteins. However, recent analysis of the lipopeptide synthetases suggests that there are several variants of NRPS architecture. Modification of NRPS by genetic engineering of the encoding genes is a promising method to produce useful variants. Accumulation of genetic information for lipopeptide synthetases should contribute to design biosurfactants with higher surface activity and/or novel features. Moreover, understanding of their biosynthetic pathways and genetic regulation mechanisms will facilitate not only uncovering the evolution of nonribosomal peptide synthesis mechanisms, but also the development of cost-effective methods for large-scale production of useful lipopeptides.

## Figures and Tables

**Table 1:** Primary structure of representative lipopeptides produced by *Bacillus* strains.

Name	Structure	Ref.
**Surfactin family**		
Surfactin	FA-β-OH-l-Glu-l-Leu-d-Leu-l-Val-l-Asp-d-Leu-l-Leu	[31]
Lichenysin	A/D FA-β-OH-l-Gln-l-Leu-d-Leu-l-Val-l-Asp-d-Leu-l-Ile	[7,32]
(Lichenysin B)	FA-β-OH-l-Glu-l-Leu-d-Leu-l-Val-l-Asp-d-Leu-l-Leu	[33]
(Lichenysin C)	FA-β-OH-l-Glu-l-Leu-d-Leu-l-Val-l-Asp-d-Leu-l-Ile	[34]
Lichenysin	G FA-β-OH-l-Gln-l-[A_2_]-d-Leu-l-[A_4_]-l-Asp-d-Leu-l-[A_7_] A_2_ = Leu/Ile, A_4_ = Val/Ile, A_7_ = Ile/Val	[6]
Surfactant BL86	FA-β-OH-l-Glx-l-Leu-d-Leu-l-Val-l-Asx-d-Leu-l-[A_7_] A_7_ = Ile/Val	[35]
Pumilacidin	FA-β-OH-l-Glu-l-Leu-d-Leu-l-Leu-l-Asp-d-Leu-l-[A_7_] A_7_ = Ile/Val	[36]
**Fengycin family**		
Fengycin	FA-β-OH-l-Glu-d-Orn-d-Tyr-d-*a*Thr-l-Glu-d-[A_6_]-l-Pro-l-Gln-l-Tyr-l-Ile A_6_ = Ala/Val	[37]
Plipastatin	FA-β-OH-l-Glu-D-Orn-l-Tyr-D-*a*Thr-l-Glu-D-[A_6_]-l-Pro-l-Gln-D-Tyr-l-Ile A_6_ = Ala/Val	[37]
**Iturin family**		
Iturin A	FA-β-NH_2_-l-Asn-d-Tyr-d-Asn-l-Gln-l-Pro-d-Asn-l-Ser	[38]
Iturin C	FA-β-NH_2_-l-Asp-d-Tyr-d-Asn-l-Gln-l-Pro-d-Asn-l-Ser	[37]
Bacillomycin L	FA-β-NH_2_-l-Asn-d-Tyr-d-Asn-l-Ser-l-Glu-d-Ser-l-Thr	[39]
Bacillomycin D	FA-β-NH_2_-l-Asn-d-Tyr-d-Asn-l-Pro-l-Glu-d-Ser-l-Thr	[37]
Bacillomycin F	FA-β-NH_2_-l-Asn-d-Tyr-d-Asn-l-Gln-l-Pro-d-Asn-l-Thr	[37]
Mycosubtilin	FA-β-NH_2_-l-Asn-d-Tyr-d-Asn-l-Gln-l-Pro-d-Ser-l-Asn	[40]
**Other**		
Antiadhesin	FA-β-OH-l-Asp-l-Leu-l-Leu-l-Val-l-Val-l-Glu-l-Leu	[41]
Bamylocin A	FA-β-OH-x-Glu-x-Leu-x-Met-x-Leu-x-Pro-x-Leu-x-Leu	[42]
Circulocin 1	*g*FA-β-OH-x-Thr-x-Phe-x-Ile-x-Dba-x-Asp	[43]
Circulocin 3	*g*FA-β-OH-x-Thr-x-Leu-x-Ile-x-Thr-x-Asn-x-Ala	[43]
Fusaricidin	*g*FA-β-OH-l-Thr-d-Val-l-Tyr-d-*a*Thr-d-Asn-d-Ala	[44]
Kurstakins	FA-x-Thr-x-Gly-x-Ala-x-Ser-x-His-x-Gln-x-Gln	[45]

**Table 2:** Primary structure of representative lipopeptides produced by *Pseudomonas.*

Group/Name	Structure	Ref.
**Viscosin**^A^		
Viscosin	FA-β-OH-l-Leu-d-Glu-d-*a*Thr-d-Val-l-Leu-d-Ser-l-Leu-d-Ser-l-Ile	[46]
Viscosinamide	FA-β-OH-l-Leu-d-Gln-d-*a*Thr-d-Val-l-Leu-d-Ser-l-Leu-d-Ser-l-Ile	[47]
Massetolide A	FA-β-OH-l-Leu-d-Glu-d-*a*Thr-d-*a*Ile-l-Leu-d-Ser-l-Leu-d-Ser-l-Ile	[48]
Pseudophomin A	FA-β-OH-l-Leu-d-Glu-d-*a*Thr-d- Ile-d-Leu-d-Ser-l-Leu-d-Ser-l-Ile	[49]
Pseudodesmin A	FA-β-OH-l-Leu-d-Gln-d-*a*Thr-d-Val-d-Leu-d-Ser-l-Leu-d-Ser-l-Ile	[50]
**Syringomycin**^B^		
Syringomycin A	FA-β-OH-l-Ser-d-Ser-d-Dab-l-Dab-l-Arg-l-Phe-z-Dhb-l-Asp_(3-OH)_-l-Thr_(4-Cl)_	[51]
Syringostatin A	FA-β-OH-l-Ser-d-Dab-l-Dab-d-Hse-l-Orn-l-*a*Thr-z-Dhb-l-Asp_(3-OH)_-l-Thr_(4-Cl)_	[52]
Syringotoxin B	FA-β-OH-l-Ser-d-Dab-l-Gly-d-Hse-l-Orn-l-*a*Thr-z-Dhb-l-Asp_(3-OH)_-l-Thr_(4-Cl)_	[53]
Pseudomycin A	FA-β-OH-l-Ser-d-Dab-l-Asp-d-Lys-l-Dab-l-*a*Thr-z-Dhb-l-Asp_(3-OH)_-l-Thr_(4-Cl)_	[54]
Cormycin A	FA-β-OH-l-Ser-d-Orn-l-Asn-d-Hse-l-His-l-*a*Thr-z-Dhb-l-Asp_(3-OH)_-l-Thr_(4-Cl)_	[55]
**Amphisin**^A^		
Arthrofactin	FA-β-OH-d-Leu-d-Asp-d-*a*Thr-d-Leu-d-Leu-d-Ser-l-Leu-d-Ser-l-Ile-l-Ile-l-Asp	[56]
Amphisin	FA-β-OH-d-Leu-d-Asp-d-*a*Thr-d-Leu-d-Leu-d-Ser-l-Leu-d-Gln-l-Leu-l-Ile-l-Asp	[10]
Lokisin	FA-β-OH-d-Leu-d-Asp-d-*a*Thr-d-Leu-d-Leu-d-Ser-l-Leu-d-Ser-l-Leu-l-Ile-l-Asp	[11]
Pholipeptin	FA-β-OH-d-Leu-l-Asp-l- Thr-d-Leu-d-Leu-d-Ser-D-leu-d-Ser-d-Leu-l-Ile-d-Asp	[12]
Tensin	FA-β-OH-d-Leu-d-Asp-d-*a*Thr-d-Leu-d-Leu-d-Ser-l-Leu-d-Gln-l-Leu-l-Ile-l-Glu	[57]
**Putisolvin**^C^		
Putisolvin I	CH_3_(CH)_4_CO-d-Leu-d-Glu-d-Leu-d-Ile-d-Gln-d-Ser-d-Val-d-Ile-d-Ser-l-Leu-l-Val-x-Ser	[58]
Putisolvin II	CH_3_(CH)_4_CO-d-Leu-d-Glu-d-Leu-d-Ile-d-Gln-d-Ser-d-Val-d-Ile-d-Ser-l-Leu-l-Xle-x-Ser	[58]
**Tolaasin**^A^		
Tolaasin I	FA-β-OH-Dhb-Pro-Ser-Leu-Val-Ser-Leu-Val-Val-Gln-Leu - - - - Val-Dhb-*a*Thr-Ile-Hse-Dab-Lys	[59]
Fuscopeptin	FA-β-OH-Dhb-Pro-Leu-Ala-Ala-Ala-Ala-Val-Gly-Ala-Val-Ala - - - Val-Dhb-*a*Thr-Ala-Dab-Dab-Phe	[60]
Corpeptin	FA-β-OH-Dhb-Pro-Ala-Ala-Ala-Val-Val-Dhb-Hse-Val-aIle-Dhb-Ala-Ala-Ala-Val-Dhb-*a*Thr-Ala-Dab-Ser-Ile	[61]
**Syringopeptin**^A^		
SP22	FA-β-OH-Dhb-Pro-Val-Val-Ala-Ala-Val - - - Val-Dhb-Ala-Val-Ala-Ala-Dhb-*a*Thr-Ser-Ala-Dhb-Ala-Dab-Dab-Tyr	[62]
SP25	FA-β-OH-Dhb-Pro-Val-Ala-Ala-Val-Leu-Ala-Ala-Dhb-Val-Dhb-Ala-Val-Ala-Ala-Dhb-*a*Thr-Ser-Ala-Val-Ala-Dab-Dab-Tyr	[63]
SP25[Phe_25_]	FA-β-OH-Dhb-Pro-Val-Ala-Ala-Val-Leu-Ala-Ala-Dhb-Val-Dhb-Ala-Val-Ala-Ala-Dhb-*a*Thr-Ser-Ala-Val-Ala-Dab-Dab-Phe	[64]
**Other**		
Entolysin^D^	FA-β-OH-d-Xle-d-Glu-d-Gln-d-Val-d-Xle-d-Gln-d-Val-d-Xle-d-Gln-d-Ser-l-Val-l-Xle-d-Ser-x-Xle	[65]
Ofamide^A^	FA-β-OH-l-Leu-d-Glu-d-*a*Thr-d-*a*Ile-l-Leu-d-Ser-l-Leu-l-Leu-d-Ser-l-Val	[66]
Pseudofactin^A^	CH_3_(CH)_14_CO-x-Gly-x-Ser-x-Thr-x-Leu-x-Leu-x-Ser-x-Leu-x-Leu/Val	[67]
Syringafactin^E^	FA-β-OH-d-Leu-d-Leu-d-Gln-l-Leu-d-Thr-l-Val-d-Leu-l-Leu	[68]

A The hydroxyl group of *a*Thr or Thr form an ester bond with the carboxyl group of the *C*-terminal amino acid

B The hydroxyl group of l-Ser_1_ form an ester bond with the carboxyl group of the *C*-terminal amino acid

C The hydroxyl group of d-Ser_9_ form an ester bond with the carboxyl group of the *C*-terminal amino acid

D The hydroxyl group of d-Ser_10_ form an ester bond with the carboxyl group of the *C*-terminal amino acid

E Linear lipopeptide

**Table 3:** Primary structure of engineered LPBSs produced by *B. subtilis.*

Name	Structure	Ref.
**Cyclic products**		
Surfactin[Val_7_]	FA-β-OH-l-Glu-l-Leu-d-Leu-l-Val-l-Asp-d-Leu-l-Val	[14]
Surfactin[Phe_7_]	FA-β-OH-l-Glu-l-Leu-d-Leu-l-Val-l-Asp-d-Leu-l-Phe	[14]
Surfactin[Orn_7_]	FA-β-OH-l-Glu-l-Leu-d-Leu-l-Val-l-Asp-d-Leu-l-Orn	[14]
Surfactin[Cys_7_]	FA-β-OH-l-Glu-l-Leu-d-Leu-l-Val-l-Asp-d-Leu-l-Cys	[14]
Surfactin[Gln_1_]	FA-β-OH-l-Gln-l-Leu-d-Leu-l-Val-l-Asp-d-Leu-l-Leu	[155]
Surfactin[Asn_5_]	FA-β-OH-l-Glu-l-Leu-d-Leu-l-Val-l-Asn-d-Leu-l-Leu	[13]
Surfactin[Orn_2_]	FA-β-OH-l-Glu-l-Orn-d-Leu-l-Val-l-Asp-d-Leu-l-Leu	[154]
Surfactin[Orn_2_ΔLeu_3_/Val_4_]	FA-β-OH-l-Glu-l-Orn-l-Asp-d-Leu-l-Leu	[154]
Surfactin[Orn_2_ΔLeu_6/7_]	FA-β-OH-l-Glu-l-Orn-d-Leu-l-Val-l-Asp	[154]
Surfactin[ΔLeu_2_]	FA-β-OH-l-Glu-d-Leu-l-Val-l-Asp-d-Leu-l-Leu	[161,162]
**Linear products**		
Surfactin[ΔLeu_7_]	FA-β-OH-l-Glu-l-Leu-d-Leu-l-Val-l-Asp-d-Leu	[14]
Surfactin[ΔLeu_6/7_]	FA-β-OH-l-Glu-l-Leu-d-Leu-l-Val-l-Asp	[156]
Surfactin[ΔAsp_5_Leu_6/7_]	FA-β-OH-l-Glu-l-Leu-d-Leu-l-Val	[156]
Surfactin[ΔVal_4_Asp_5_Leu_6_]	FA-β-OH-l-Glu-l-Leu-d-Leu-l-Leu	[165]

## References

[1] Georgiou G Lin SC Sharma MM Surface-active compounds from microorganisms Biotechnology (NY) 1992 10 60 65 10.1038/nbt0192-60 1368190

[2] Desai JD Banat IM Microbial production of surfactants and their commercial potential Microbiol. Mol. Biol. Rev 1997 61 47 64 9106364 10.1128/mmbr.61.1.47-64.1997 PMC232600

[3] Arima K Kakinuma A Tamura G Surfactin, a crystalline peptidelipid surfactant produced by *Bacillus subtilis*: isolation, characterization and its inhibition of fibrin clot formation Biochem. Biophys. Res. Commun 1968 31 488 494 4968234 10.1016/0006-291x(68)90503-2

[4] Nishikiori T Naganawa H Muraoka Y Aoyagi T Umezawa H Plipastatins: new inhibitors of phospholipase A2, produced by *Bacillus cereus* BMG302-fF67. III. Structural elucidation of plipastatins J. Antibiot (Tokyo) 1986 39 755 761 3089999 10.7164/antibiotics.39.755

[5] Peypoux F Pommier MT Das BC Besson F Delcambe L Michel G Structures of bacillomycin D and bacillomycin L peptidolipid antibiotics from *Bacillus subtilis* J. Antibiot (Tokyo) 1984 37 1600 1604 6441791 10.7164/antibiotics.37.1600

[6] Grangemard I Bonmatin JM Bernillon J Das BC Peypoux F Lichenysins G, a novel family of lipopeptide biosurfactants from *Bacillus licheniformis* IM 1307: production, isolation and structural evaluation by NMR and mass spectrometry J. Antibiot (Tokyo) 1999 52 363 373 10395272 10.7164/antibiotics.52.363

[7] Yakimov MM Abraham WR Meyer H Laura G Golyshin PN Structural characterization of lichenysin A components by fast atom bombardment tandem mass spectrometry Biochim. Biophys Acta 1999 1438 273 280 10320810 10.1016/s1388-1981(99)00058-x

[8] Morikawa M Hirata Y Imanaka T A study on the structure-function relationship of lipopeptide biosurfactants Biochim. Biophys Acta 2000 1488 211 218 11082531 10.1016/s1388-1981(00)00124-4

[9] Nielsen TH Sorensen D Tobiasen C Andersen JB Christophersen C Givskov M Sorensen J Antibiotic and biosurfactant properties of cyclic lipopeptides produced by fluorescent *Pseudomonas* spp. from the sugar beet rhizosphere Appl. Environ. Microbiol 2002 68 3416 3423 12089023 10.1128/AEM.68.7.3416-3423.2002 PMC126818

[10] Sorensen D Nielsen TH Christophersen C Sorensen J Gajhede M Cyclic lipoundecapeptide amphisin from *Pseudomonas* sp. strain DSS73 Acta Crystallogr C 2001 57 1123 1124 11588392 10.1107/s0108270101010782

[11] Sorensen D Nielsen TH Sorensen J Christophersen C Cyclic lipoundecapeptide lokisin from *Pseudomonas* sp. strain DSS41 Tetrahedron Lett 2002 43 4421 4423

[12] Ui H Miyake T Iinuma H Imoto M Naganawa H Hattori S Hamada M Takeuchi T Umezawa S Umezawa K Pholipeptin, a novel cyclic lipoundecapeptide from *Pseudomonas fluorescens* J. Org. Chem 1997 62 103 108 11671369 10.1021/jo9603993

[13] Eppelmann K Stachelhaus T Marahiel MA Exploitation of the selectivity-conferring code of nonribosomal peptide synthetases for the rational design of novel peptide antibiotics Biochemistry 2002 41 9718 9726 12135394 10.1021/bi0259406

[14] Stachelhaus T Schneider A Marahiel MA Rational design of peptide antibiotics by targeted replacement of bacterial and fungal domains Science 1995 269 69 72 7604280 10.1126/science.7604280

[15] Koglin A Walsh CT Structural insights into nonribosomal peptide enzymatic assembly lines Nat. Prod. Rep 2009 26 987 1000 19636447 10.1039/b904543k PMC2773127

[16] Raaijmakers JM de Bruijn I Nybroe O Ongena M Natural functions of lipopeptides from *Bacillus* and *Pseudomonas*: more than surfactants and antibiotics FEMS Microbiol Rev 2010 10.1111/j.1574-6976.2010.00221.x 20412310

[17] Roongsawang N Thaniyavarn J Thaniyavarn S Kameyama T Haruki M Imanaka T Morikawa M Kanaya S Isolation and characterization of a halotolerant *Bacillus subtilis* BBK-1 which produces three kinds of lipopeptides: bacillomycin L, plipastatin, and surfactin Extremophiles 2002 6 499 506 12486459 10.1007/s00792-002-0287-2

[18] Koumoutsi A Chen XH Henne A Liesegang H Hitzeroth G Franke P Vater J Borriss R Structural and functional characterization of gene clusters directing nonribosomal synthesis of bioactive cyclic lipopeptides in *Bacillus amyloliquefaciens* strain FZB42 J. Bacteriol 2004 186 1084 1096 14762003 10.1128/JB.186.4.1084-1096.2004 PMC344220

[19] Romero D de Vicente A Rakotoaly RH Dufour SE Veening JW Arrebola E Cazorla FM Kuipers OP Paquot M Perez-Garcia A The iturin and fengycin families of lipopeptides are key factors in antagonism of *Bacillus subtilis* toward *Podosphaera fusca* Mol. Plant Microbe Interact 2007 20 430 440 17427813 10.1094/MPMI-20-4-0430

[20] Kim PI Ryu J Kim YH Chi YT Production of biosurfactant lipopeptides iturin A, fengycin and surfactin A from *Bacillus subtilis* CMB32 for control of *Colletotrichum gloeosporioides* J. Microbiol. Biotechnol 2010 20 138 145 20134245

[21] Yakimov MM Timmis KN Wray V Fredrickson HL Characterization of a new lipopeptide surfactant produced by thermotolerant and halotolerant subsurface *Bacillus licheniformis* BAS50 Appl. Environ. Microbiol 1995 61 1706 1713 7646007 10.1128/aem.61.5.1706-1713.1995 PMC167432

[22] Bonmatin JM Laprevote O Peypoux F Diversity among microbial cyclic lipopeptides: iturins and surfactins. Activity-structure relationships to design new bioactive agents Comb. Chem High Throughput Screen 2003 6 541 556 14529379 10.2174/138620703106298716

[23] Grangemard I Wallach J Maget-Dana R Peypoux F Lichenysin: a more efficient cation chelator than surfactin Appl. Biochem. Biotechnol 2001 90 199 210 11318033 10.1385/abab:90:3:199

[24] Rodrigues L Banat IM Teixeira J Oliveira R Biosurfactants: potential applications in medicine J. Antimicrob. Chemother 2006 57 609 618 16469849 10.1093/jac/dkl024

[25] Bais HP Fall R Vivanco JM Biocontrol of *Bacillus subtilis* against infection of Arabidopsis roots by *Pseudomonas syringae* is facilitated by biofilm formation and surfactin production Plant Physiol 2004 134 307 319 14684838 10.1104/pp.103.028712 PMC316310

[26] Hofemeister J Conrad B Adler B Hofemeister B Feesche J Kucheryava N Steinborn G Franke P Grammel N Zwintscher A Leenders F Hitzeroth G Vater J Genetic analysis of the biosynthesis of nonribosomal peptide and polyketide-like antibiotics, iron uptake and biofilm formation by *Bacillus subtilis* A1/3 Mol. Genet Genomics 2004 272 363 378 15480790 10.1007/s00438-004-1056-y

[27] Kearns DB Losick R Swarming motility in undomesticated *Bacillus subtilis* Mol. Microbiol 2003 49 581 590 12864845 10.1046/j.1365-2958.2003.03584.x

[28] Julkowska D Obuchowski M Holland IB Seror SJ Comparative analysis of the development of swarming communities of *Bacillus subtilis* 168 and a natural wild type: critical effects of surfactin and the composition of the medium J. Bacteriol 2005 187 65 76 15601689 10.1128/JB.187.1.65-76.2005 PMC538812

[29] Branda SS Gonzalez-Pastor JE Ben-Yehuda S Losick R Kolter R Fruiting body formation by *Bacillus subtilis* Proc. Natl. Acad. Sci USA 2001 98 11621 11626 11572999 10.1073/pnas.191384198 PMC58779

[30] Mireles JR Toguchi A Harshey RM *Salmonella enterica* serovar *typhimurium* swarming mutants with altered biofilm-forming abilities: surfactin inhibits biofilm formation J. Bacteriol 2001 183 5848 5854 11566982 10.1128/JB.183.20.5848-5854.2001 PMC99661

[31] Kakinuma A Ouchida A Shima T Sugino H Isono M Tamura G Arima K Conformation of the structure of surfactin by mass spectrometry Agric. Biol. Chem 1969 33 1669 1671

[32] Konz D Doekel S Marahiel MA Molecular and biochemical characterization of the protein template controlling biosynthesis of the lipopeptide lichenysin J. Bacteriol 1999 181 133 140 9864322 10.1128/jb.181.1.133-140.1999 PMC103541

[33] Lin SC Minton MA Sharma MM Georgiou G Structural and immunological characterization of a biosurfactant produced by *Bacillus licheniformis* JF-2 Appl. Environ. Microbiol 1994 60 31 38 8117083 10.1128/aem.60.1.31-38.1994 PMC201265

[34] Jenny K Kappeli O Fiechter A Biosurfactants from *Bacillus licheniformis*: structural analysis and characterization Appl. Microbiol. Biotechnol 1991 36 5 13 1367776 10.1007/BF00164690

[35] Horowitz S Gilbert JN Griffin WM Isolation and characterization of a surfactant produced by *Bacillus licheniformis* 86 J. Indus. Microbiol 1990 6 243 248

[36] Naruse N Tenmyo O Kobaru S Kamei H Miyaki T Konishi M Oki T Pumilacidin, a complex of new antiviral antibiotics. Production, isolation, chemical properties, structure and biological activity J Antibiot (Tokyo) 1990 43 267 280 2157695 10.7164/antibiotics.43.267

[37] Volpon L Besson F Lancelin JM NMR structure of antibiotics plipastatins A and B from *Bacillus subtilis* inhibitors of phospholipase A(2) FEBS Lett 2000 485 76 80 11086169 10.1016/s0014-5793(00)02182-7

[38] Isogai I Takayama S Murakoshi S Suzuki A Structure of β-amino acids in antibiotics iturin A Tetrahedron Lett 1982 23 3065 3068

[39] Volpon L Tsan P Majer Z Vass E Hollosi M Noguera V Lancelin JM Besson F NMR structure determination of a synthetic analogue of bacillomycin Lc reveals the strategic role of L-Asn1 in the natural iturinic antibiotics Spectrochim Acta Part A 2007 67 1374 1381 10.1016/j.saa.2006.10.027 17129757

[40] Duitman EH Hamoen LW Rembold M Venema G Seitz H Saenger W Bernhard F Reinhardt R Schmidt M Ullrich C Stein T Leenders F Vater J The mycosubtilin synthetase of *Bacillus subtilis* ATCC6633: a multifunctional hybrid between a peptide synthetase, an amino transferase, and a fatty acid synthase Proc. Natl. Acad. Sci USA 1999 96 13294 13299 10557314 10.1073/pnas.96.23.13294 PMC23941

[41] Batrakov SG Rodionova TA Esipov SE Polyakov NB Sheichenko VI Shekhovtsova NV Lukin SM Panikov NS Nikolaev YA A novel lipopeptide, an inhibitor of bacterial adhesion, from the thermophilic and halotolerant subsurface *Bacillus licheniformis* strain 603 Biochim. Biophys Acta 2003 1634 107 115 14643798 10.1016/j.bbalip.2003.09.004

[42] Lee SC Kim SH Park IH Chung SY Choi YL Isolation and structural analysis of bamylocin A, novel lipopeptide from *Bacillus amyloliquefaciens* LP03 having antagonistic and crude oil-emulsifying activity Arch. Microbiol 2007 188 307 312 17530228 10.1007/s00203-007-0250-9

[43] He H Shen B Korshalla J Carter GT Circulocins, new antibacterial lipopeptides from *Bacillus circulans*, J2154 Tetrahedron 2001 57 1189 1195

[44] Li J Jensen SE Nonribosomal biosynthesis of fusaricidins by *Paenibacillus polymyxa* PKB1 involves direct activation of a D-amino acid Chem. Biol 2008 15 118 127 18291316 10.1016/j.chembiol.2007.12.014

[45] Hathout Y Ho YP Ryzhov V Demirev P Fenselau C Kurstakins: a new class of lipopeptides isolated from *Bacillus thuringiensis* J. Nat. Prod 2000 63 1492 1496 11087590 10.1021/np000169q

[46] de Bruijn I de Kock MJ Yang M de Waard P van Beek TA Raaijmakers JM Genome-based discovery, structure prediction and functional analysis of cyclic lipopeptide antibiotics in *Pseudomonas* species Mol. Microbiol 2007 63 417 428 17241198 10.1111/j.1365-2958.2006.05525.x

[47] Nielsen TH Christophersen C Anthoni U Sorensen J Viscosinamide, a new cyclic depsipeptide with surfactant and antifungal properties produced by *Pseudomonas fluorescens* DR54 J. Appl. Microbiol 1999 87 80 90 10432590 10.1046/j.1365-2672.1999.00798.x

[48] de Bruijn I de Kock MJ de Waard P van Beek TA Raaijmakers JM Massetolide A biosynthesis in *Pseudomonas fluorescens* J. Bacteriol 2008 190 2777 2789 17993540 10.1128/JB.01563-07 PMC2293227

[49] Quail JW Ismail N Pedras MS Boyetchko SM Pseudophomins A and B, a class of cyclic lipodepsipeptides isolated from a *Pseudomonas* species Acta Crystallogr C 2002 58 268 271 10.1107/s0108270102004432 11983987

[50] Sinnaeve D Hendrickx PM Van Hemel J Peys E Kieffer B Martins JC The solution structure and self-association properties of the cyclic lipodepsipeptide pseudodesmin A support its pore-forming potential Chemistry 2009 15 12653 12662 19839018 10.1002/chem.200901885

[51] Guenzi E Galli G Grgurina I Gross DC Grandi G Characterization of the syringomycin synthetase gene cluster. A link between prokaryotic and eukaryotic peptide synthetases J. Biol. Chem 1998 273 32857 32863 9830033 10.1074/jbc.273.49.32857

[52] Sorensen KN Kim KH Takemoto JY *In vitro* antifungal and fungicidal activities and erythrocyte toxicities of cyclic lipodepsinonapeptides produced by *Pseudomonas syringae* pv. syringae Antimicrob. Agents Chemother 1996 40 2710 2713 9124827 10.1128/aac.40.12.2710 PMC163608

[53] Ballio A Bossa F Collina A Gallo M Iacobellis NS Paci M Pucci P Scaloni A Segre A Simmaco M Structure of syringotoxin, a bioactive metabolite of *Pseudomonas syringae* pv. syringae FEBS Lett 1990 269 377 380 2401362 10.1016/0014-5793(90)81197-v

[54] Harrison L Teplow DB Rinaldi M Strobel G Pseudomycins, a family of novel peptides from *Pseudomonas syringae* possessing broad-spectrum antifungal activity J. Gen. Microbiol 1991 137 2857 2865 1791440 10.1099/00221287-137-12-2857

[55] Scaloni A Dalla Serra M Amodeo P Mannina L Vitale RM Segre AL Cruciani O Lodovichetti F Greco ML Fiore A Gallo M D’Ambrosio C Coraiola M Menestrina G Graniti A Fogliano V Structure, conformation and biological activity of a novel lipodepsipeptide from *Pseudomonas corrugata*: cormycin A Biochem. J 2004 384 25 36 15196052 10.1042/BJ20040422 PMC1134085

[56] Morikawa M Daido H Takao T Murata S Shimonishi Y Imanaka T A new lipopeptide biosurfactant produced by *Arthrobacter* sp. strain MIS38 J. Bacteriol 1993 175 6459 6466 8407822 10.1128/jb.175.20.6459-6466.1993 PMC206754

[57] Henriksen A Anthoni U Nielsen TH Sorensen J Christophersen C Gajhede M Cyclic lipoundecapeptide tensin from *Pseudomonas fluorescens* strain 96.578 Acta Crystallogr C 2000 56 Pt 1 113 115 10710691 10.1107/s0108270199013414

[58] Kuiper I Lagendijk EL Pickford R Derrick JP Lamers GE Thomas-Oates JE Lugtenberg BJ Bloemberg GV Characterization of two *Pseudomonas putida* lipopeptide biosurfactants, putisolvin I and II, which inhibit biofilm formation and break down existing biofilms Mol. Microbiol 2004 51 97 113 14651614 10.1046/j.1365-2958.2003.03751.x

[59] Bassarello C Lazzaroni S Bifulco G Lo Cantore P Iacobellis NS Riccio R Gomez- Paloma L Evidente A Tolaasins A-E, five new lipodepsipeptides produced by *Pseudomonas tolaasii* J. Nat. Prod 2004 67 811 816 15165142 10.1021/np0303557

[60] Ballio A Bossa F Camoni L Di Giorgio D Flamand MC Maraite H Nitti G Pucci P Scaloni A Structure of fuscopeptins, phytotoxic metabolites of *Pseudomonas fuscovaginae* FEBS Lett 1996 381 213 216 8601458 10.1016/0014-5793(96)00043-9

[61] Emanuele MC Scaloni A Lavermicocca P Jacobellis NS Camoni L Di Giorgio D Pucci P Paci M Segre A Ballio A Corpeptins, new bioactive lipodepsipeptides from cultures of *Pseudomonas corrugata* FEBS Lett 1998 433 317 320 9744818 10.1016/s0014-5793(98)00933-8

[62] Ballio A Barra D Bossa F Collina A Grgurina I Marino G Moneti G Paci M Pucci P Segre A Syringopeptins, new phytotoxic lipodepsipeptides of *Pseudomonas syringae* pv. syringae FEBS Lett 1991 291 109 112 1936237 10.1016/0014-5793(91)81115-o

[63] Ballio A Bossa F Di Giorgio D Di Nola A Manetti C Paci M Scaloni A Segre AL Solution conformation of the *Pseudomonas syringae* pv. *syringae* phytotoxic lipodepsipeptide syringopeptin 25-A. Two-dimensional NMR, distance geometry and molecular dynamics Eur. J. Biochem 1995 234 747 758 8575431 10.1111/j.1432-1033.1995.747_a.x

[64] Scaloni A Camoni L di Giorgio D Scortichini M Cozzolino R Ballio A A new syringopeptin produced by a *Pseudomonas syringae* pv *syringae* strain isolated from diseased twigs of laurel Physiol. Mol. Plant Pathol 1997 51 259 264

[65] Vallet-Gely I Novikov A Augusto L Liehl P Bolbach G Pechy-Tarr M Cosson P Keel C Caroff M Lemaitre B Association of hemolytic activity of *Pseudomonas entomophila*, a versatile soil bacterium, with cyclic lipopeptide production Appl. Environ. Microbiol 2010 76 910 921 20023108 10.1128/AEM.02112-09 PMC2812987

[66] Gross H Stockwell VO Henkels MD Nowak-Thompson B Loper JE Gerwick WH The genomisotopic approach: a systematic method to isolate products of orphan biosynthetic gene clusters Chem. Biol 2007 14 53 63 17254952 10.1016/j.chembiol.2006.11.007

[67] Janek T Lukaszewicz M Rezanka T Krasowska A Isolation and characterization of two new lipopeptide biosurfactants produced by *Pseudomonas fluorescens* BD5 isolated from water from the Arctic Archipelago of Svalbard Bioresour. Technol 2010 101 6118 6123 20303744 10.1016/j.biortech.2010.02.109

[68] Berti AD Greve NJ Christensen QH Thomas MG Identification of a biosynthetic gene cluster and the six associated lipopeptides involved in swarming motility of *Pseudomonas syringae* pv. *tomato* DC3000 J. Bacteriol 2007 189 6312 6323 17601782 10.1128/JB.00725-07 PMC1951903

[69] Cosmina P Rodriguez F de Ferra F Grandi G Perego M Venema G van Sinderen D Sequence and analysis of the genetic locus responsible for surfactin synthesis in *Bacillus subtilis* Mol. Microbiol 1993 8 821 831 8355609 10.1111/j.1365-2958.1993.tb01629.x

[70] Roongsawang N Lim SP Washio K Takano K Kanaya S Morikawa M Phylogenetic analysis of condensation domains in the nonribosomal peptide synthetases FEMS Microbiol. Lett 2005 252 143 151 16182472 10.1016/j.femsle.2005.08.041

[71] Kraas FI Helmetag V Wittmann M Strieker M Marahiel MA Functional dissection of surfactin synthetase initiation module reveals insights into the mechanism of lipoinitiation Chem. Biol 2010 17 872 880 20797616 10.1016/j.chembiol.2010.06.015

[72] Schneider A Marahiel MA Genetic evidence for a role of thioesterase domains, integrated in or associated with peptide synthetases, in non-ribosomal peptide biosynthesis in *Bacillus subtilis* Arch. Microbiol 1998 169 404 410 9560421 10.1007/s002030050590

[73] Schwarzer D Mootz HD Linne U Marahiel MA Regeneration of misprimed nonribosomal peptide synthetases by type II thioesterases Proc. Natl. Acad. Sci USA 2002 99 14083 14088 12384573 10.1073/pnas.212382199 PMC137840

[74] Yeh E Kohli RM Bruner SD Walsh CT Type II thioesterase restores activity of a NRPS module stalled with an aminoacyl-S-enzyme that cannot be elongated ChemBioChem 2004 5 1290 1293 15368584 10.1002/cbic.200400077

[75] Menkhaus M Ullrich C Kluge B Vater J Vollenbroich D Kamp RM Structural and functional organization of the surfactin synthetase multienzyme system J. Biol. Chem 1993 268 7678 7684 8096516

[76] Steller S Sokoll A Wilde C Bernhard F Franke P Vater J Initiation of surfactin biosynthesis and the role of the SrfD-thioesterase protein Biochemistry 2004 43 11331 11343 15366943 10.1021/bi0493416

[77] Vanittanakom N Loeffler W Koch U Jung G Fengycin-a novel antifungal lipopeptide antibiotic produced by *Bacillus subtilis* F-29-3 J. Antibiot (Tokyo) 1986 39 888 901 3093430 10.7164/antibiotics.39.888

[78] Rivardo F Turner RJ Allegrone G Ceri H Martinotti MG Anti-adhesion activity of two biosurfactants produced by *Bacillus* spp. prevents biofilm formation of human bacterial pathogens Appl. Microbiol. Biotechnol 2009 83 541 553 19343338 10.1007/s00253-009-1987-7

[79] Thaniyavarn J Roongsawang N Kameyama T Haruki M Imanaka T Morikawa M Kanaya S Production and characterization of biosurfactants from *Bacillus licheniformis* F2.2 Biosci. Biotechnol. Biochem 2003 67 1239 1244 12843648 10.1271/bbb.67.1239

[80] Wu CY Chen CL Lee YH Cheng YC Wu YC Shu HY Gotz F Liu ST Nonribosomal synthesis of fengycin on an enzyme complex formed by fengycin synthetases J. Biol. Chem 2007 282 5608 5616 17182617 10.1074/jbc.M609726200

[81] Leclere V Marti R Bechet M Fickers P Jacques P The lipopeptides mycosubtilin and surfactin enhance spreading of *Bacillus subtilis* strains by their surface-active properties Arch. Microbiol 2006 186 475 483 16964493 10.1007/s00203-006-0163-z

[82] Tsuge K Akiyama T Shoda M Cloning, sequencing, and characterization of the iturin A operon J. Bacteriol 2001 183 6265 6273 11591669 10.1128/JB.183.21.6265-6273.2001 PMC100110

[83] Moyne AL Cleveland TE Tuzun S Molecular characterization and analysis of the operon encoding the antifungal lipopeptide bacillomycin D FEMS Microbiol. Lett 2004 234 43 49 15109718 10.1016/j.femsle.2004.03.011

[84] Hansen DB Bumpus SB Aron ZD Kelleher NL Walsh CT The loading module of mycosubtilin: an adenylation domain with fatty acid selectivity J. Am. Chem. Soc 2007 129 6366 6367 17472382 10.1021/ja070890j

[85] Aron ZD Fortin PD Calderone CT Walsh CT FenF: servicing the mycosubtilin synthetase assembly line in trans ChemBioChem 2007 8 613 616 17330903 10.1002/cbic.200600575

[86] Aron ZD Dorrestein PC Blackhall JR Kelleher NL Walsh CT Characterization of a new tailoring domain in polyketide biogenesis: the amine transferase domain of MycA in the mycosubtilin gene cluster J. Am. Chem. Soc 2005 127 14986 14987 16248612 10.1021/ja055247g

[87] Dittmann J Wenger RM Kleinkauf H Lawen A Mechanism of cyclosporin A biosynthesis. Evidence for synthesis via a single linear undecapeptide precursor J. Biol. Chem 1994 269 2841 2846 8300618

[88] Walton JD Two enzymes involved in biosynthesis of the host-selective phytotoxin HC-toxin Proc. Natl. Acad. Sci USA 1987 84 8444 8447 16593904 10.1073/pnas.84.23.8444 PMC299560

[89] Balibar CJ Vaillancourt FH Walsh CT Generation of D amino acid residues in assembly of arthrofactin by dual condensation/epimerization domains Chem. Biol 2005 12 1189 1200 16298298 10.1016/j.chembiol.2005.08.010

[90] Gross H Loper JE Genomics of secondary metabolite production by *Pseudomonas* spp Nat. Prod. Rep 2009 26 1408 1446 19844639 10.1039/b817075b

[91] Hutchison ML Gross DC Lipopeptide phytotoxins produced by *Pseudomonas syringae* pv. *syringae*: comparison of the biosurfactant and ion channel-forming activities of syringopeptin and syringomycin Mol. Plant Microbe Interact 1997 10 347 354 9100379 10.1094/MPMI.1997.10.3.347

[92] Guenzi E Galli G Grgurina I Pace E Ferranti P Grandi G Coordinate transcription and physical linkage of domains in surfactin synthetase are not essential for proper assembly and activity of the multienzyme complex J. Biol. Chem 1998 273 14403 14410 9603952 10.1074/jbc.273.23.14403

[93] Vaillancourt FH Yin J Walsh CT SyrB2 in syringomycin E biosynthesis is a nonheme FeII alpha-ketoglutarate- and O_2_-dependent halogenase Proc. Natl. Acad. Sci USA 2005 102 10111 10116 16002467 10.1073/pnas.0504412102 PMC1177402

[94] Singh GM Vaillancourt FH Yin J Walsh CT Characterization of SyrC, an aminoacyltransferase shuttling threonyl and chlorothreonyl residues in the syringomycin biosynthetic assembly line Chem. Biol 2007 14 31 40 17254950 10.1016/j.chembiol.2006.11.005

[95] Singh GM Fortin PD Koglin A Walsh CT *beta*-Hydroxylation of the aspartyl residue in the phytotoxin syringomycin E: characterization of two candidate hydroxylases AspH and SyrP in *Pseudomonas syringae* Biochemistry 2008 47 11310 11320 18826255 10.1021/bi801322z PMC2600472

[96] Scholz-Schroeder BK Soule JD Gross DC The *sypA*, *sypB*, and *sypC* synthetase genes encode twenty-two modules involved in the nonribosomal peptide synthesis of syringopeptin by *Pseudomonas syringae* pv. *syringae* B301D Mol. Plant Microbe Interact 2003 16 271 280 12744455 10.1094/MPMI.2003.16.4.271

[97] Ikegami T Osaka University Osaka, Japan Personal communication 2004

[98] Roongsawang N Hase K Haruki M Imanaka T Morikawa M Kanaya S Cloning and characterization of the gene cluster encoding arthrofactin synthetase from *Pseudomonas* sp. MIS38 Chem. Biol 2003 10 869 880 14522057 10.1016/j.chembiol.2003.09.004

[99] Andersen JB Koch B Nielsen TH Sorensen D Hansen M Nybroe O Christophersen C Sorensen J Molin S Givskov M Surface motility in *Pseudomonas* sp. DSS73 is required for efficient biological containment of the root-pathogenic microfungi *Rhizoctonia solani* and *Pythium ultimum* Microbiology 2003 149 37 46 12576578 10.1099/mic.0.25859-0

[100] Roongsawang N Washio K Morikawa M Hokkaido University Hokkaido, Japan Unpublished work 2007

[101] Roongsawang N Washio K Morikawa M *In vivo* characterization of tandem *C*-terminal thioesterase domains in arthrofactin synthetase ChemBioChem 2007 8 501 512 17328008 10.1002/cbic.200600465

[102] Lim SP Roongsawang N Washio K Morikawa M Functional analysis of a pyoverdine synthetase from *Pseudomonas* sp. MIS38 Biosci. Biotechnol. Biochem 2007 71 2002 2009 17690457 10.1271/bbb.70185

[103] Saini HS Barragan-Huerta BE Lebron-Paler A Pemberton JE Vazquez RR Burns AM Marron MT Seliga CJ Gunatilaka AA Maier RM Efficient purification of the biosurfactant viscosin from *Pseudomonas libanensis* strain M9-3 and its physicochemical and biological properties J. Nat. Prod 2008 71 1011 1015 18471020 10.1021/np800069u

[104] Kruijt M Tran H Raaijmakers JM Functional, genetic and chemical characterization of biosurfactants produced by plant growth-promoting *Pseudomonas putida* 267 J. Appl. Microbiol 2009 107 546 556 19302489 10.1111/j.1365-2672.2009.04244.x

[105] Dubern JF Coppoolse ER Stiekema WJ Bloemberg GV Genetic and functional characterization of the gene cluster directing the biosynthesis of putisolvin I and II in *Pseudomonas putida* strain PCL1445 Microbiology 2008 154 2070 2083 18599835 10.1099/mic.0.2008/016444-0

[106] Magnuson R Solomon J Grossman AD Biochemical and genetic characterization of a competence pheromone from *Bacillus subtilis* Cell 1994 77 207 216 8168130 10.1016/0092-8674(94)90313-1

[107] Auchtung JM Lee CA Grossman AD Modulation of the ComA-dependent quorum response in *Bacillus subtilis* by multiple Rap proteins and Phr peptides J. Bacteriol 2006 188 5273 5285 16816200 10.1128/JB.00300-06 PMC1539962

[108] Roggiani M Dubnau D ComA, a phosphorylated response regulator protein of *Bacillus subtilis*, binds to the promoter region of *srfA* J. Bacteriol 1993 175 3182 3187 8387999 10.1128/jb.175.10.3182-3187.1993 PMC204641

[109] Yakimov MM Golyshin PN ComA-dependent transcriptional activation of lichenysin A synthetase promoter in *Bacillus subtilis* cells Biotechnol. Prog 1997 13 757 761 9413133 10.1021/bp9700622

[110] Griffith KL Grossman AD A degenerate tripartite DNA-binding site required for activation of ComA-dependent quorum response gene expression in *Bacillus subtilis* J. Mol. Biol 2008 381 261 275 18585392 10.1016/j.jmb.2008.06.035 PMC2604127

[111] Wang X Luo C Liu Y Nie Y Zhang R Chen Z Three non-aspartate amino acid mutations in the ComA Response regulator receiver motif severely decrease surfactin production, competence development and spore formation in *Bacillus subtilis* J. Microbiol. Biotechnol 2010 20 301 310 20208433

[112] Nakano MM Zuber P Cloning and characterization of *srfB*, a regulatory gene involved in surfactin production and competence in *Bacillus subtilis* J. Bacteriol 1989 171 5347 5353 2507521 10.1128/jb.171.10.5347-5353.1989 PMC210372

[113] Kim SB Shin BS Choi SK Kim CK Park SH Involvement of acetyl phosphate in the *in vivo* activation of the response regulator ComA in *Bacillus subtilis* FEMS Microbiol. Lett 2001 195 179 183 11179649 10.1111/j.1574-6968.2001.tb10518.x

[114] Cosby WM Vollenbroich D Lee OH Zuber P Altered srf expression in *Bacillus subtilis* resulting from changes in culture pH is dependent on the Spo0K oligopeptide permease and the ComQX system of extracellular control J. Bacteriol 1998 180 1438 1445 9515911 10.1128/jb.180.6.1438-1445.1998 PMC107042

[115] Hayashi K Ohsawa T Kobayashi K Ogasawara N Ogura M The H_2_O_2_ stress-responsive regulator PerR positively regulates *srfA* expression in *Bacillus subtilis* J. Bacteriol 2005 187 6659 6667 16166527 10.1128/JB.187.19.6659-6667.2005 PMC1251593

[116] Nakano MM Zhu Y Liu J Reyes DY Yoshikawa H Zuber P Mutations conferring amino acid residue substitutions in the carboxy-terminal domain of RNA polymerase alpha can suppress *clpX* and *clpP* with respect to developmentally regulated transcription in *Bacillus subtilis* Mol. Microbiol 2000 37 869 884 10972808 10.1046/j.1365-2958.2000.02052.x

[117] Mader U Antelmann H Buder T Dahl MK Hecker M Homuth G *Bacillus subtilis* functional genomics: genome-wide analysis of the DegS-DegU regulon by transcriptomics and proteomics Mol. Genet Genomics 2002 268 455 467 12471443 10.1007/s00438-002-0774-2

[118] Hayashi K Kensuke T Kobayashi K Ogasawara N Ogura M *Bacillus subtilis* RghR (YvaN) represses *rapG* and *rapH*, which encode inhibitors of expression of the *srfA* operon Mol. Microbiol 2006 59 1714 1729 16553878 10.1111/j.1365-2958.2006.05059.x

[119] Ogura M Fujita Y *Bacillus subtilis rapD*, a direct target of transcription repression by RghR, negatively regulates *srfA* expression FEMS Microbiol. Lett 2007 268 73 80 17227471 10.1111/j.1574-6968.2006.00559.x

[120] Ohsawa T Tsukahara K Sato T Ogura M Superoxide stress decreases expression of *srfA* through inhibition of transcription of the *comQXP* quorum-sensing locus in *Bacillus subtilis* J. Biochem 2006 139 203 211 16452308 10.1093/jb/mvj023

[121] Serror P Sonenshein AL CodY is required for nutritional repression of *Bacillus subtilis* genetic competence J. Bacteriol 1996 178 5910 5915 8830686 10.1128/jb.178.20.5910-5915.1996 PMC178446

[122] Nakano S Nakano MM Zhang Y Leelakriangsak M Zuber P A regulatory protein that interferes with activator-stimulated transcription in bacteria Proc. Natl. Acad. Sci USA 2003 100 4233 4238 12642660 10.1073/pnas.0637648100 PMC153076

[123] Reyes DY Zuber P Activation of transcription initiation by Spx: formation of transcription complex and identification of a *cis*-acting element required for transcriptional activation Mol. Microbiol 2008 69 765 779 18687074 10.1111/j.1365-2958.2008.06330.x PMC2758557

[124] Quadri LE Weinreb PH Lei M Nakano MM Zuber P Walsh CT Characterization of Sfp, a *Bacillus subtilis* phosphopantetheinyl transferase for peptidyl carrier protein domains in peptide synthetases Biochemistry 1998 37 1585 1595 9484229 10.1021/bi9719861

[125] Tsuge K Ohata Y Shoda M Gene *yerP*, involved in surfactin self-resistance in *Bacillus subtilis* Antimicrob. Agents Chemother 2001 45 3566 3573 11709341 10.1128/AAC.45.12.3566-3573.2001 PMC90870

[126] Tsuge K Ano T Hirai M Nakamura Y Shoda M The genes *degQ*, *pps*, and *lpa-8* (*sfp*) are responsible for conversion of *Bacillus subtilis* 168 to plipastatin production Antimicrob. Agents Chemother 1999 43 2183 2192 10471562 10.1128/aac.43.9.2183 PMC89444

[127] Msadek T Kunst F Klier A Rapoport G DegS-DegU and ComP-ComA modulator-effector pairs control expression of the *Bacillus subtilis* pleiotropic regulatory gene *degQ* J. Bacteriol 1991 173 2366 2377 1901055 10.1128/jb.173.7.2366-2377.1991 PMC207789

[128] Tsuge K Ano T Shoda M Isolation of a gene essential for biosynthesis of the lipopeptide antibiotics plipastatin B1 and surfactin in *Bacillus subtilis* YB8 Arch. Microbiol 1996 165 243 251 8639027 10.1007/s002030050322

[129] Ke WJ Chang BY Lin TP Liu ST Activation of the promoter of the fengycin synthetase operon by the UP element J. Bacteriol 2009 191 4615 4623 19447911 10.1128/JB.00255-09 PMC2704709

[130] Duitman EH Wyczawski D Boven LG Venema G Kuipers OP Hamoen LW Novel methods for genetic transformation of natural *Bacillus subtilis* isolates used to study the regulation of the mycosubtilin and surfactin synthetases Appl. Environ. Microbiol 2007 73 3490 3496 17416694 10.1128/AEM.02751-06 PMC1932663

[131] Koumoutsi A Chen XH Vater J Borriss R DegU and YczE positively regulate the synthesis of bacillomycin D by *Bacillus amyloliquefaciens* strain FZB42 Appl. Environ. Microbiol 2007 73 6953 6964 17827323 10.1128/AEM.00565-07 PMC2074971

[132] Huang CC Ano T Shoda M Nucleotide sequence and characteristics of the gene, lpa-14, responsible for biosynthesis of the lipopeptide antibiotics iturinA and surfactin from *Bacillus subtilis* RB14 J. Ferment. Bioeng 1993 76 445 450

[133] Heeb S Haas D Regulatory roles of the GacS/GacA two-component system in plant-associated and other gram-negative bacteria Mol. Plant Microbe Interact 2001 14 1351 1363 11768529 10.1094/MPMI.2001.14.12.1351

[134] Workentine ML Chang L Ceri H Turner RJ The GacS-GacA two-component regulatory system of *Pseudomonas fluorescens*: a bacterial two-hybrid analysis FEMS Microbiol. Lett 2009 292 50 56 19191877 10.1111/j.1574-6968.2008.01445.x

[135] Willis DK Holmstadt JJ Kinscherf TG Genetic evidence that loss of virulence associated with *gacS* or *gacA* mutations in *Pseudomonas syringae* B728a does not result from effects on alginate production Appl. Environ. Microbiol 2001 67 1400 1403 11229941 10.1128/AEM.67.3.1400-1403.2001 PMC92744

[136] Koch B Nielsen TH Sorensen D Andersen JB Christophersen C Molin S Givskov M Sorensen J Nybroe O Lipopeptide production in *Pseudomonas* sp. strain DSS73 is regulated by components of sugar beet seed exudate via the Gac two-component regulatory system Appl. Environ. Microbiol 2002 68 4509 4516 12200307 10.1128/AEM.68.9.4509-4516.2002 PMC124083

[137] Nielsen TH Nybroe O Koch B Hansen M Sorensen J Genes involved in cyclic lipopeptide production are important for seed and straw colonization by *Pseudomonas* sp. strain DSS73 Appl. Environ. Microbiol 2005 71 4112 4116 16000829 10.1128/AEM.71.7.4112-4116.2005 PMC1169020

[138] Dubern JF Bloemberg GV Influence of environmental conditions on putisolvins I and II production in *Pseudomonas putida* strain PCL1445 FEMS Microbiol. Lett 2006 263 169 175 16978352 10.1111/j.1574-6968.2006.00406.x

[139] Dubern JF Lugtenberg BJ Bloemberg GV The *ppuI*-*rsaL*-*ppuR* quorum-sensing system regulates biofilm formation of *Pseudomonas putida* PCL1445 by controlling biosynthesis of the cyclic lipopeptides putisolvins I and II J. Bacteriol 2006 188 2898 2906 16585751 10.1128/JB.188.8.2898-2906.2006 PMC1447005

[140] Washio K Lim SP Roongsawang N Morikawa M Identification and characterization of the genes responsible for the production of the cyclic lipopeptide arthrofactin by *Pseudomonas* sp. MIS38 Biosci. Biotechnol. Biochem 2010 74 992 999 20460722 10.1271/bbb.90860

[141] Dubern JF Lagendijk EL Lugtenberg BJ Bloemberg GV The heat shock genes *dnaK*, *dnaJ*, and *grpE* are involved in regulation of putisolvin biosynthesis in *Pseudomonas putida* PCL1445 J. Bacteriol 2005 187 5967 5976 16109938 10.1128/JB.187.17.5967-5976.2005 PMC1196155

[142] Wang N Lu SE Records AR Gross DC Characterization of the transcriptional activators SalA and SyrF, Which are required for syringomycin and syringopeptin production by *Pseudomonas syringae* pv. syringae J. Bacteriol 2006 188 3290 3298 16621822 10.1128/JB.188.9.3290-3298.2006 PMC1447436

[143] de Bruijn I Raaijmakers JM Diversity and functional analysis of LuxR-type transcriptional regulators of cyclic lipopeptide biosynthesis in *Pseudomonas fluorescens* Appl. Environ. Microbiol 2009 75 4753 4761 19447950 10.1128/AEM.00575-09 PMC2708414

[144] de Bruijn I Raaijmakers JM Regulation of cyclic lipopeptide biosynthesis in *Pseudomonas fluorescens* by the ClpP protease J. Bacteriol 2009 191 1910 1923 19114474 10.1128/JB.01558-08 PMC2648375

[145] Srivatsan A Wang JD Control of bacterial transcription, translation and replication by (p)ppGpp Curr. Opin. Microbiol 2008 11 100 105 18359660 10.1016/j.mib.2008.02.001

[146] Finking R Solsbacher J Konz D Schobert M Schafer A Jahn D Marahiel MA Characterization of a new type of phosphopantetheinyl transferase for fatty acid and siderophore synthesis in *Pseudomonas aeruginosa* J. Biol. Chem 2002 277 50293 50302 12381736 10.1074/jbc.M205042200

[147] Barekzi N Joshi S Irwin S Ontl T Schweizer HP Genetic characterization of *pcpS*, encoding the multifunctional phosphopantetheinyl transferase of *Pseudomonas aeruginosa* Microbiology 2004 150 795 803 15073290 10.1099/mic.0.26823-0

[148] Seidle HF Couch RD Parry RJ Characterization of a nonspecific phosphopantetheinyl transferase from *Pseudomonas syringae* pv. *syringae* FF5 Arch. Biochem. Biophys 2006 446 167 174 16423321 10.1016/j.abb.2005.12.007

[149] Quigley NB Mo YY Gross DC SyrD is required for syringomycin production by *Pseudomonas syringae* pv. *syringae* and is related to a family of ATP-binding secretion proteins Mol. Microbiol 1993 9 787 801 8231810 10.1111/j.1365-2958.1993.tb01738.x

[150] Grgurina I Gross DC Iacobellis NS Lavermicocca P Takemoto JY Benincasa M Phytotoxin production by *Pseudomonas syringae* pv. *syringae*: Syringopeptin production by *syr* mutants defective in biosynthesis or secretion of syringomycin FEMS Microbiol. Lett 1996 138 35 39

[151] Kang H Gross DC Characterization of a resistance-nodulation-cell division transporter system associated with the *syr*-*syp* genomic island of *Pseudomonas syringae* pv. syringae Appl. Environ. Microbiol 2005 71 5056 5065 16151087 10.1128/AEM.71.9.5056-5065.2005 PMC1214623

[152] Lim SP Roongsawang N Washio K Morikawa M Flexible exportation mechanisms of arthrofactin in *Pseudomonas* sp. MIS38 J. Appl. Microbiol 2009 107 157 166 19302333 10.1111/j.1365-2672.2009.04189.x

[153] Kobayashi N Nishino K Yamaguchi A Novel macrolide-specific ABC-type efflux transporter in *Escherichia coli* J. Bacteriol 2001 183 5639 5644 11544226 10.1128/JB.183.19.5639-5644.2001 PMC95455

[154] Schneider A Stachelhaus T Marahiel MA Targeted alteration of the substrate specificity of peptide synthetases by rational module swapping Mol. Gen. Genet 1998 257 308 318 9520265 10.1007/s004380050652

[155] Yakimov MM Giuliano L Timmis KN Golyshin PN Recombinant acylheptapeptide lichenysin: high level of production by *Bacillus subtilis* cells J. Mol. Microbiol. Biotechnol 2000 2 217 224 10939247

[156] de Ferra F Rodriguez F Tortora O Tosi C Grandi G Engineering of peptide synthetases. Key role of the thioesterase-like domain for efficient production of recombinant peptides J. Biol. Chem 1997 272 25304 25309 9312148 10.1074/jbc.272.40.25304

[157] Trauger JW Kohli RM Mootz HD Marahiel MA Walsh CT Peptide cyclization catalysed by the thioesterase domain of tyrocidine synthetase Nature 2000 407 215 218 11001063 10.1038/35025116

[158] Kohli RM Trauger JW Schwarzer D Marahiel MA Walsh CT Generality of peptide cyclization catalyzed by isolated thioesterase domains of nonribosomal peptide synthetases Biochemistry 2001 40 7099 7108 11401555 10.1021/bi010036j

[159] Stachelhaus T Mootz HD Marahiel MA The specificity-conferring code of adenylation domains in nonribosomal peptide synthetases Chem. Biol 1999 6 493 505 10421756 10.1016/S1074-5521(99)80082-9

[160] Uguru GC Milne C Borg M Flett F Smith CP Micklefield J Active-site modifications of adenylation domains lead to hydrolysis of upstream nonribosomal peptidyl thioester intermediates J. Am. Chem. Soc 2004 126 5032 5033 15099062 10.1021/ja048778y

[161] Mootz HD Kessler N Linne U Eppelmann K Schwarzer D Marahiel MA Decreasing the ring size of a cyclic nonribosomal peptide antibiotic by in-frame module deletion in the biosynthetic genes J. Am. Chem. Soc 2002 124 10980 10981 12224936 10.1021/ja027276m

[162] Symmank H Franke P Saenger W Bernhard F Modification of biologically active peptides: production of a novel lipohexapeptide after engineering of *Bacillus subtilis* surfactin synthetase Protein Eng 2002 15 913 921 12538911 10.1093/protein/15.11.913

[163] Butz D Schmiederer T Hadatsch B Wohlleben W Weber T Sussmuth RD Module extension of a non-ribosomal peptide synthetase of the glycopeptide antibiotic balhimycin produced by *Amycolatopsis balhimycina* ChemBioChem 2008 9 1195 1200 18399534 10.1002/cbic.200800068

[164] Hahn M Stachelhaus T Harnessing the potential of communication-mediating domains for the biocombinatorial synthesis of nonribosomal peptides Proc. Natl. Acad. Sci USA 2006 103 275 280 16407157 10.1073/pnas.0508409103 PMC1326170

[165] Chiocchini C Linne U Stachelhaus T *In vivo* biocombinatorial synthesis of lipopeptides by COM domain-mediated reprogramming of the surfactin biosynthetic complex Chem. Biol 2006 13 899 908 16931339 10.1016/j.chembiol.2006.06.015

[166] Vater J Wilde C Kell H *In situ* detection of the intermediates in the biosynthesis of surfactin, a lipoheptapeptide from *Bacillus subtilis* OKB 105, by whole-cell cell matrix-assisted laser desorption/ionization time-of-flight mass spectrometry in combination with mutant analysis Rapid. Commun. Mass Spectrom 2009 23 1493 1498 19350532 10.1002/rcm.4031

[167] Ackerley DF Lamont IL Characterization and genetic manipulation of peptide synthetases in *Pseudomonas aeruginosa* PAO1 in order to generate novel pyoverdines Chem. Biol 2004 11 971 980 15271355 10.1016/j.chembiol.2004.04.014

[168] Baltz RH Biosynthesis and genetic engineering of lipopeptides in *Streptomyces roseosporus* Methods Enzymol 2009 458 511 531 19374996 10.1016/S0076-6879(09)04820-4

[169] Nguyen KT He X Alexander DC Li C Gu JQ Mascio C Van Praagh A Mortin L Chu M Silverman JA Brian P Baltz RH Genetically engineered lipopeptide antibiotics related to A54145 and daptomycin with improved properties Antimicrob. Agents Chemother 2010 54 1404 1413 20086142 10.1128/AAC.01307-09 PMC2849371

